# Early Nephrology Consultation and Acute Kidney Injury in Hospitalized Patients: A Randomized Clinical Trial

**DOI:** 10.1001/jamanetworkopen.2026.22554

**Published:** 2026-07-10

**Authors:** Matthew M. Churpek, Aiman Fatima, Olasunkanmi Anjorin, Ananya Saravanan, Benjamin S. Ko, Samantha Gunning, Megan L. Prochaska, Tipu S. Puri, Anna L. Zisman, Dana P. Edelson, Mihai C. Giurcanu, Jay L. Koyner

**Affiliations:** 1 Department of Medicine, University of Wisconsin–Madison; 2 Department of Biostatistics and Medical Informatics, University of Wisconsin–Madison; 3 Section of Nephrology, Department of Medicine, University of Chicago, Chicago, Illinois; 4 Department of Medicine, University of Chicago, Chicago, Illinois; 5 Committee on Clinical Pharmacology and Pharmacogenomics, University of Chicago, Chicago, Illinois; 6 Department of Public Health Sciences, University of Chicago, Chicago, Illinois

## Abstract

**Objective:**

To determine whether a structured early nephrology consultation triggered by a machine-learning acute kidney injury (AKI) risk score (electronic signal to prevent AKI [ESTOP-AKI]) in patients at high risk for stage 2 AKI improves patient outcomes.

**Design, Setting, and Participants:**

This randomized clinical trial was conducted at the University of Chicago, Illinois, from March 13, 2019, to August 21, 2024, in hospitalized patients with no serum creatinine (SCr)–based AKI and an ESTOP-AKI score more than 0.01.

**Intervention:**

Patients were randomized to receive a structured early nephrology consultation (ENC) from an attending nephrologist or usual care (UC). The ENC included an in-person assessment and recommendations regarding volume status, kidney perfusion, medication dosing and selection, electrolytes, nutritional needs, and further testing. Patients in the UC arm only received a nephrology consultation when clinically requested by the primary team.

**Main Outcomes and Measures:**

The primary outcome was the peak change in SCr from enrollment (ΔSCr) during the 7-day follow-up. Secondary outcomes included development of AKI, need for kidney replacement therapy, and inpatient and 90-day mortality.

**Results:**

Of the 180 patients randomized (median [IQR] age, 62.5 [50.0-71.0] years; 102 males [56.7%]), 89 (49.4%) received ENC, and 91 (50.6%) received UC. There was no significant adjusted mean (SE) difference in the 7-day ΔSCr between the ENC and UC groups, adjusted for the ESTOP risk group (0.04 [0.07] mg/dL vs −0.03 [0.07] mg/dL; *P* = .30), among the 70 patients (38.9%) in the development of stage 1 or higher AKI (37 [42%] vs 33 [36%]; *P* = .47) or among the 29 patients (16.1%) with stage 2 or higher AKI (17 [19%] vs 12 [13%]; *P* = .28). During the study period, there were 121 ENC consultations containing 270 recommendations compared with 19 UC consultations and 36 recommendations. Medication dosage and discontinuation, diuretics or fluids, and vasopressor recommendations were more likely to be completely followed in the UC arm (15 of 22 [68%]) compared with in the ENC arm (48 of 116 [41%]). Over the 90-day follow-up, there was no significant difference in readmission rates (ENC: 30 [34.1%] vs UC: 40 [44.4%]; *P* = .21) or 90-day mortality (ENC: 13 [14.8%] vs UC: 17 [18.7%]; *P* = .62) between the ENC and UC arms.

**Conclusions and Relevance:**

In this randomized clinical trial of structured ENC triggered by a machine-learning AKI risk score, there was no difference in ΔSCr. AKI consultation recommendations were not followed the majority of time; whether increasing adherence to recommendations could improve outcomes deserves further study.

**Trial Registration:**

ClinicalTrials.gov Identifier: NCT03590028

## Key Points

**Question:**

Among inpatients at risk for severe acute kidney injury (AKI), does an early structured nephrology consultation, triggered by a machine-learning risk score, lead to smaller peak changes in serum creatinine (SCr)?

**Findings:**

In this randomized clinical trial of 180 patients at high risk for AKI, an early nephrology consultation triggered by a machine-learning model did not reduce the peak change in SCr. Consultation recommendations were less likely to be followed for patients in the early consultation arm compared with the usual care arm.

**Meaning:**

The findings of this study suggest that early nephrology consultations do not prevent rises in peak SCr among hospitalized patients at risk for severe AKI.

## Teaser

This randomized clinical trial assesses whether a structured early nephrology consultation triggered by a machine-learning acute kidney injury (AKI) risk score in patients at high risk for AKI improves patient outcomes.

## Introduction

Acute kidney injury (AKI), defined as an increase in serum creatinine (SCr) or a decrease in urine output, is a common clinical syndrome in hospitalized patients and is associated with increased costs, morbidity, and mortality.^1,2,3,4,5^ Earlier identification and intervention may improve patient outcomes in the setting of inpatient AKI.^6,7,8,9^ To our knowledge, prior clinical trials that have intervened after the development of SCr-based AKI have failed to show consistent prevention in severe AKI, need for kidney replacement therapy, or other patient outcomes.^7,8,10,11^ However, none of these randomized clinical trials (RCTs), to date, have solely targeted patients prior to the development of AKI.

AKI risk scores have shown promise in identifying patients before the development of SCr-based AKI.^12,13,14,15,16,17^ Dynamic risk scores that continually assess AKI risk throughout a hospital admission have been developed using machine-learning methods, which could prompt earlier intervention. Our group developed a gradient boosted machine-learning AKI risk score (electronic signal to prevent AKI [ESTOP-AKI]) that accurately predicts the future development of stage 2 AKI, with higher scores indicating an increased risk of developing stage 2 AKI, and was externally validated in a separate health system.^12,13^ One potential use of these models would be to trigger kidney-focused care bundles, which have been shown to improve patient outcomes for those at increased risk of AKI or with early-stage AKI.^6,8,18^ However, whether combining machine-learning AKI scores with expert-driven care recommendations improves patient outcomes remains unclear.^12,14,17^ To address this gap in the literature, we performed an RCT to determine whether a structured, proactive early nephrology consultation (ENC) triggered by an elevated ESTOP-AKI risk score in patients without SCr-based AKI could minimize future peaks in SCr, prevent the development of AKI, and improve other short-term and long-term patient-centered outcomes.

## Methods

### Study Overview

This single-center RCT was approved by the University of Chicago Institutional Review Board. The trial was performed in accordance with the Declaration of Helsinki^26^ and all other applicable regulatory requirements. The trial protocol can be found in Supplement 1. Consecutive sampling enrollment occurred between March 13, 2019, and April 1, 2020, and then from August 22, 2022, to August 21, 2024. Enrollment was paused due to the COVID-19 pandemic between these dates. Written informed consent was obtained for all participants. The trial followed the Consolidated Standards of Reporting Trials (CONSORT) reporting guideline.

### Inclusion and Exclusion Criteria

Adult patients (aged ≥18 years) hospitalized at the University of Chicago Medicine who had an ESTOP-AKI model score of at least 0.01 within the prior 8 hours without any evidence of Kidney Disease: Improving Global Outcomes (KDIGO) SCr-based AKI during admission, were eligible for enrollment. This cutoff was chosen to optimize enrollment opportunities as well as to ensure inclusion of those with an increased risk of KDIGO AKI. The ESTOP-AKI score was calculated in real-time based on patients’ laboratory and vital sign results and their most recent changes in selected values as previously described.^12^ Admission, discharge, transfer, laboratory, and flow sheet data interfaces were sent with Health Level 7, version 2 (Health Level Seven International) to a cloud-based predictive scoring engine (AgileMD) and were written back to the electronic medical record (Epic) as flow sheet observations. The ESTOP flow sheet was reported on a real-time dashboard for all hospitalized patients, visible to the research team (A.F., O.A., A.S., and J.L.K.). Research assistants (A.F., O.A., and A.S.) screened patients using this dashboard, and patients were considered eligible if they had an elevated (≥0.01) ESTOP-AKI score in the prior 8 hours and did not meet KDIGO SCr-AKI criteria. Baseline SCr was calculated as the median (IQR) value from all available values prior to admission from the past 6 months; if no prior values were available, then the first admission SCr was deemed to be the baseline. Race categories included American Indian or Alaska Native, Asian, Black, White, and other (multiracial and unknown), and the ethnicity category included Hispanic. Race and ethnicity were ascertained by the electronic medical records and were included in the study because Black race has been consistently associated with higher incidence of AKI compared with White race. We excluded patients with a known history of end-stage kidney disease or kidney transplant, without an SCr measurement during the inpatient stay, with a prior episode of SCr-based AKI or an SCr more than 4.0 mg/dL during admission, with a current diagnosis of COVID-19, or with a prior kidney consultation for any reason during their current admission.

### Randomization

Patients were randomized 1:1 to either ENC or usual care (UC) using a permuted block randomization in block sizes of 4, 6, or 8 in a REDCap module. Randomization was stratified based on patient location at enrollment (intensive care unit [ICU] vs ward) and peak ESTOP-AKI score prior to enrollment (0.01-0.0569 vs ≥0.057). The research team enrolling patients did not have access to the randomization allocation sequence.

### Intervention

The intervention was a structured, in-person ENC and formatted note that was placed in the patient’s medical record; the nephrologist spoke with the patient’s inpatient care team within 6 hours of randomization (eTable 1 in Supplement 2). Research consultations were performed by 1 of 5 nephrology attendings (B.S.K., S.G., M.L.P., T.S.P., or A.L.Z.), who were educated and provided feedback around the structured consultation contents prior to trial initiation. The consultation consisted of recommendations around 6 domains: volume status, kidney perfusion, medication selection and dosing, electrolytes, nutritional needs, and further testing. While only 1 research-based consultation was mandated per protocol, follow-up research consultations in the ENC arm were allowed if there were issues on which to follow up but required a full structured follow-up consultation, mirroring the original consultation. If patients in the ENC required kidney replacement therapy (KRT), then they no longer received research consultations and had a traditional (unstructured) nonresearch consultation. Patients in the UC arm did not receive a nephrology consultation for AKI unless there was SCr-based AKI and then would only receive a traditional nonresearch, unstructured nephrology consultation. ENC-based recommendations were not entered as orders, were treated like traditional clinical consultation recommendations (as in the UC arm), and were not mandated to be implemented. Recommendations from all consultations (research and nonresearch) were recorded through manual medical record review by members of the research team and then categorized as complete, partially followed, or not followed. As an example, consider a hypothetical patient for whom the research consultation recommended that the patient receive 80 mg of intravenous furosemide. If the patient did not receive any furosemide, the recommendation would be considered not followed. However, if the inpatient care team administered 40 mg of intravenous furosemide, this recommendation would be considered partially followed.

Patients were generally enrolled in the morning and early afternoon, and in-person research consultations and the recommendations were personally reported to the primary team and entered in the electronic medical record by afternoon or early evening, consistent with usual care at the University of Chicago. If a UC patient’s team requested a nephrology consultation, the structured tool for the ENC was not used. If patients randomized to the ENC arm required KRT, a nonresearch consultation was conducted, and a nonresearch nephrologist provided the KRT care.

### Outcomes

The primary outcome of this RCT was the peak change in SCr from enrollment (ΔSCr) over the 7-day study. Patients were not required to remain in the hospital for 7 days. Secondary outcomes included development of KDIGO AKI (using SCr and urine output criteria), need for inpatient KRT, hospital length of stay, inpatient mortality, major adverse kidney events at 90 days, major adverse cardiovascular events at 90 days, and 90-day mortality.

### Adverse Events

We collected adverse events (inpatient mortality, need for ICU transfer) across both arms of the study. Additionally, the study had an independent data safety monitoring board, which was established at the start of the trial. As per the established protocol, the data safety monitoring board met 3 times: at study initiation, after a mandatory analysis when 20% to 25% of enrollment was met, and again at 50% enrollment. These latter 2 meetings (April 2023 and February 2024) were conducted to assess safety and feasibility, as well as to determine efficacy and futility based on predetermined statistical cutoffs.

### Sample Size Estimation

The sample size was determined to detect a mean difference in ΔSCr from a baseline of 0.35 mg/dL or more over the first 7 days (to convert SCr to μmol/L, multiply by 88.4). This would allow us to detect a difference in the development of KDIGO stage 1 SCr-based AKI.^5^ Using a conventional 2-sided (type I error) α = .05 and power of 80%, it was determined that to detect this 0.35 mg/dL or more ΔSCr, we needed 83 patients per group (166 total). To ensure that we met these requirements, we elected to enroll 90 patients per arm (180 total). For secondary end points, at this sample size, the study was 80% powered to detect a 62% reduction in the development of stage 2 AKI but was underpowered to detect differences across KRT and inpatient mortality.

### Statistical Analysis

We performed stratified analyses based on the inpatient location at the time of enrollment (ICU vs ward) and used the Fisher exact test if at least 1 count was 5 or less and the χ^2^ test otherwise, while continuous variables were compared using the 2-sample *t* test or Wilcoxon rank sum test, as appropriate. The primary analysis for ΔSCr was a comparison of means from a 2-factor (treatment and ESTOP group) linear model and a comparison using a 2-proportion test for binary outcomes (AKI stage, ICU transfer). Secondary analyses were based on linear and logistic regression adjusting for ESTOP group and key factors (age, sex, location, and baseline SCr). All statistical analyses were completed using R, version 4.3.3 (R Project for Statistical Computing) with a 2-sided *P* value less than .05 considered statistically significant. We performed a post hoc analysis, in which patients who developed SCr-based AKI within 6 hours of enrollment (prior to their consultation) were excluded, as these patients developed AKI before the ENC intervention.

## Results

### Trial Population

We identified 3929 patients with an elevated risk score prior to enrollment (Figure). Preexisting AKI and end-stage kidney disease were common reasons for exclusion. Among the 180 patients randomized (median [IQR] age, 62.5 [50.0-71.0] years; 78 females [43.3%] and 102 males [56.7%]), 89 (49.4%) received ENC and 91 (50.6%) received UC. Of the total patients’ race categories, 1 (0.5%) was American Indian or Alaska Native, 4 (2.2%) were Asian, 74 (41.1%) were Black, 95 (52.8%) were White, and 6 (3.3%) were of other race, and 6 patients (3.3%) were Hispanic ethnicity.

**Figure.**
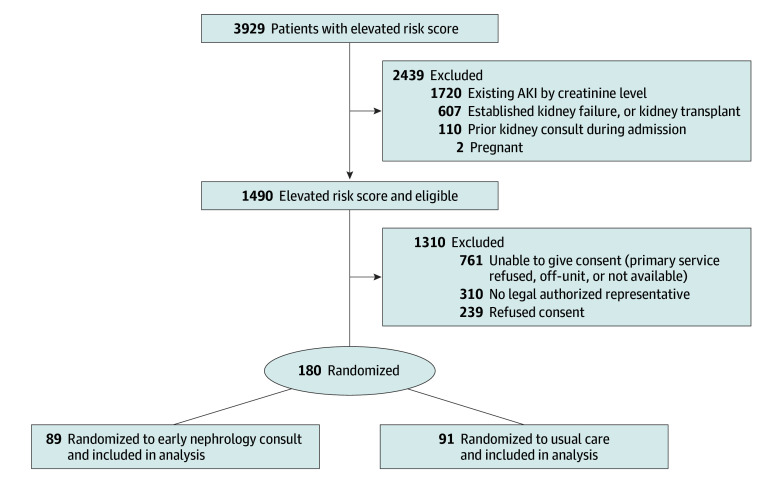
CONSORT Flow Diagram Patients were included with electronic signal to prevent (ESTOP) acute kidney injury (AKI) risk scores.

### Baseline Comparisons Between Study Arms

Clinical demographics and preenrollment data for both the ENC and UC arms are presented in Table 1. There was no significant difference in age, sex, race, or past medical history between the 2 study arms. Thirty-nine enrolled patients (21.7%) had no preadmission SCr values; they had a median (IQR) 3.0 (2.0-9.5) mg/dL inpatient values prior to enrollment to establish their baseline SCr and the absence of AKI. The other 141 patients had a median (IQR) 4.5 (2.0-11.5) mg/dL SCr values over the previous 6 months to establish a baseline SCr . Patients in both arms had similar mean (SD) preadmission baseline SCr (1.02 [0.44] mg/dL) across the entire cohort. Similarly, their mean (SD) randomization SCr values were 1.03 (0.38) mg/dL in the ENC arm and 1.10 (0.47) mg/dL in the UC arm (Table 1). There were no significant median (IQR) differences in the severity of illness scores calculated at the time of randomization for the Sequential Organ Failure Assessment score (ENC: 3.50 [2.75-5.25]; UC: 4.00 [3.00-7.00]), in which scores range from 0 to 24, with higher scores indicating more severe illness and greater risk for ICU mortality; the Acute Physiology and Chronic Health Evaluation II (ENC: 15.00 [12.00-20.00]; UC: 17.50 [14.25-21.00]), in which scores range from 0 to 71, with higher scores indicating severity of illness and greater risk for ICU mortality; and the Modified Early Warning Score (ENC: 3.00 [2.00-4.00]; UC: 3.00 [2.00-4.75]), in which scores range from 0 to 14, with higher scores indicating greater risk of clinical deterioration of a patient on the hospital ward (non-ICU). Patients in the UC arm were not more likely to have a urinary catheter in place at the time of randomization (51 [56%] vs 37 [41.6%]; *P* = .08) and were not statistically significantly less likely to have diabetes (18 [19.8%] vs 29 [32.6%]; *P* = .07). eTables 2 and 3 in Supplement 2 present the baseline comparisons between study arms stratified by location at time of randomization. Patients on the hospital ward were more likely to have a higher median (IQR) ESTOP score at enrollment (*P* = .02), but not to have a higher median (IQR) different maximum ESTOP score prior to enrollment (*P* = .14).

**Table 1.** Baseline Demographics and Characteristics of Enrolled Patients Overall and Grouped by Treatment Arm^a^

Characteristic	Overall (N = 180)	ENC (n = 89)	UC (n = 91)
Age, median (IQR), y	62.50 (50.00-71.00)	59.00 (50.00-70.00)	65.00 (53.00-72.50)
Sex			
Female	78 (43.3)	36 (40.4)	42 (46.2)
Male	102 (56.7)	53 (59.6)	49 (53.8)
Race			
American Indian or Alaska Native	1 (0.5)	1 (1.1)	0
Asian	4 (2.2)	3 (3.3)	1 (1.1)
Black	74 (41.1)	38 (42.7)	36 (39.6)
White	95 (52.8)	43 (48.3)	52 (57.1)
Other^b^	6 (3.3)	4 (4.5)	2 (2.2)
Hispanic ethnicity	6 (3.3)	4 (4.5)	2 (2.2)
Past medical history			
Hypertension	104 (57.8)	50 (56.2)	54 (59.3)
Diabetes	47 (26.1)	29 (32.6)	18 (19.8)
Chronic kidney disease	12 (6.7)	5 (5.6)	7 (7.7)
Congestive heart failure	23 (12.8)	12 (13.5)	11 (12.1)
Cancer	46 (25.6)	22 (24.7)	24 (26.4)
ESTOP at enrollment, median (IQR)	0.01 (0.01-0.02)	0.01 (0.01-0.02)	0.01 (0.01-0.02)
Maximum ESTOP before enrollment, median (IQR)	0.02 (0.01-0.03)	0.02 (0.01-0.03)	0.02 (0.01-0.03)
ESTOP risk group			
High (≥0.057)	16 (8.9)	9 (10.1)	7 (7.7)
Moderate (0.01-0.0569)	164 (91.1)	80 (89.9)	84 (92.3)
Patient location at randomization			
Ward or floor	79 (43.9)	41 (46.1)	38 (41.8)
ICU	101 (56.1)	48 (53.9)	53 (58.2)
Time from admission to enrollment, median (IQR), h	65.00 (42.00-114.42)	64.50 (41.80-90.00)	65.00 (42.10-115.40)
Mechanical ventilation at enrollment	37 (20.6)	18 (20.2)	19 (20.9)
Foley catheter at enrollment	88 (48.9)	37 (41.6)	51 (56.0)
SOFA at enrollment for patients in the ICU, median (IQR)^c^	4.00 (3.00-6.25)	3.50 (2.75-5.25)	4.00 (3.00-7.00)
MEWS at enrollment for patients on the hospital ward, median (IQR)^d^	3.00 (2.00-4.00)	3.00 (2.00-4.00)	3.00 (2.00-4.75)
APACHE II at enrollment for patients in the ICU, median (IQR)^e^	15.00 (12.00-21.00)	15.00 (12.00-20.00)	17.50 (14.25-21.00)
Prehospitalization baseline SCr, mean (SD), mg/dL	1.02 (0.44)	0.98 (0.36)	1.05 (0.50)
Enrollment SCr, mean (SD), mg/dL	1.07 (0.43)	1.03 (0.38)	1.10 (0.47)
Enrollment blood urea nitrogen, mean (SD), mg/dL	23.00 (16.70)	22.90 (15.60)	23.00 (17.90)

Abbreviations: APACHE II, Acute Physiology and Chronic Health Evaluation; ENC, early nephrology consultation; ESTOP, electronic signal to prevent (acute kidney injury); ICU, intensive care unit; MEWS, Modified Early Warning Score; SCr, serum creatinine; SOFA, Sequential Organ Failure Assessment; UC, usual care.

SI conversion factor: To convert SCr to µmol/L, multiply by 88.4; urea nitrogen to mmol/L, multiply by 0.357.

^a^ Data are reported as No. (%) unless otherwise indicated.

^b^ Includes multiracial and unknown.

^c^ Scores range from 0 to 24, with higher scores indicating more severe illness and greater risk for ICU mortality.

^d^ Scores range from 0 to 14, with higher scores indicating greater risk of clinical deterioration of a patient on the hospital ward (non-ICU).

^e^ Scores range from 0 to 71, with higher scores indicating severity of illness and greater risk for ICU mortality.

### Outcomes

Table 2 shows the study patient outcomes overall and grouped by study arms. There was no significant adjusted mean (SE) difference in the ΔSCr over the 7 days following enrollment between the ENC and UC arms (0.04 [0.07] mg/dL vs −0.03 [0.07] mg/dL; *P* = .30). The mean (SE) difference in ΔSCr over 7 days adjusted for stratification factors (ESTOP group) was not significant (0.08 [0.07] mg/dL; *P* = .30), and adjusting for age, sex, location, and baseline SCr, the difference in the adjusted mean (SE) was not significant (0.09 [0.07] mg/dL; *P* = .24). No covariates were statistically significant in the adjusted analysis. The eFigure in Supplement 2 plots the median (IQR) SCr over the first 7 days of the study for both study arms, which was not significantly different between groups. There were 9 of 89 ENC patients (10.1%) and 13 of 91 UC patients (14.3%) (*P* = .50) who had a decrease in SCr of 0.03 mg/dL or more (in the absence of preenrollment AKI) during the first 7 days. There was no difference in the development of KDIGO stage 1 or higher AKI (KDIGO ≥1) between the ENC and UC arms (37 [42%] vs 33 [36%]; *P* = .47), with 70 patients (38.9%) developing any KDIGO AKI over the 7 days after enrollment. Twenty-nine patients (16.1%) developed stage 2 or 3 AKI (KDIGO ≥2) over the 7 days, with no differences between ENC and UC arms (17 [19%] vs 12 [13%]; *P* = .28). Logistic regression analysis found that although the ENC arm did not have statistically significantly higher odds of developing any KDIGO AKI score (odds ratio [OR], 1.68 [95% CI, 0.86-3.28]; *P* = .13) or KDIGO stage 2 AKI or higher (OR, 1.78 [95% CI, 0.76-4.13]; *P* = .19). In addition, baseline SCr (OR, 4.95 [95% CI, 1.95-12.54] mg/dL; *P* = .001) and ICU location (OR, 4.00 [95% CI, 1.93-8.32]; *P* < .001) were associated with higher odds of a KDIGO score of 1 or more and ICU location with higher odds of KDIGO stage 2 AKI or higher (OR, 4.84 [95% CI, 1.67-14.03]; *P* = .004). There was no significant difference between the ENC and UC arms in the need for KRT (3 [3.4%] vs 2 [2.2%]; *P* = .98; total: 5 [2.8%]) or for the inpatient mortality rates (8 [9.0%] vs 6 [6.7%]; *P* = .75; total: 14 [7.8%]) over the course of the index hospital admission (Table 2). There was no mean (SD) difference in ΔSCr between the ENC and UC study arms stratified by enrollment location at the ICU (0.08 [0.58] mg/dL vs −0.02 [0.49] mg/dL; *P* = .37) and the ward (0.01 [0.43] mg/dL vs −0.04 [0.39] mg/dL; *P* = .60) (eTables 4 and 5 in Supplement 2).

**Table 2.** Inpatient Outcomes of Enrolled Patients Overall and Grouped by Treatment Arm

Inpatient outcome	Overall (N = 180)	ENC (n = 89)	UC (n = 91)	*P* value^a^
Peak SCr during first 7 d after enrollment, mean (SD), mg/dL	1.08 (0.67)	1.08 (0.65)	1.08 (0.69)	.98
Peak change in SCr from enrollment, mean (SD), mg/dL	0.01 (0.48)	0.05 (0.52)	−0.03 (0.45)	.30
Required KRT, No. (%)	5 (2.8)	3 (3.4)	2 (2.2)	.98
Mortality, No. (%)	14 (7.8)	8 (9.0)	6 (6.7)	.75
Maximum KDIGO stage, No. (%)				
No AKI	110 (61.1)	52 (58.4)	58 (63.7)	.75
1	41 (22.8)	20 (22.5)	21 (23.1)	
2	24 (13.3)	14 (15.7)	10 (11.0)	
3	5 (2.8)	3 (3.4)	2 (2.2)	
Duration of all AKI (n = 70), median (IQR), d	2.00 (1.00-3.00)	2.00 (1.00-3.00)	2.00 (1.00-4.50)	.58
Duration of severe AKI (n = 30), median (IQR), d	3.00 (2.00-4.00)	3.00 (2.00-4.00)	2.00 (2.00-5.75)	.99
Hospital length of stay, median (IQR), d	9.00 (5.00-16.25)	9.00 (6.00-15.00)	9.00 (5.00-17.00)	.96
ICU length of stay, median (IQR), d	3.00 (2.00-7.00)	4.00 (2.00-6.00)	3.00 (1.25-7.00)	.60

Abbreviations: AKI, acute kidney injury; ENC, early nephrology consultation; ICU, intensive care unit; KDIGO, Kidney Disease: Improving Global Outcomes; KRT, kidney replacement therapy; SCr, serum creatinine; UC, usual care.

SI conversion factor: To convert SCr to µmol/L, multiply by 88.4.

^a^ Categorical variables are compared using the χ^2^ test and the Fisher exact test if at least 1 count was 5 or less and summarized as No. (%); continuous variables are compared by the Wilcoxon rank sum test and summarized by medians (IQRs); and means (SDs) are compared between groups using the 2-sample *t* test.

Table 3 shows the 90-day follow-up outcomes by study arms. There was no significant median (IQR) difference in 90-day SCr values between the ENC and UC arms (1.10 [0.75-1.30] mg/dL vs 1.00 [0.70-1.50] mg/dL; *P* = .83). There were 70 patients (39.3%) readmitted to a hospital over the 90 days after enrollment (ENC: 30 [34.1%] vs UC: 40 [44.4%]; *P* = .21), and during these readmissions, there were no significant differences in need for nephrology consultation (ENC: 10 [33.3%] vs UC: 12 [30.0%]; *P* = .97). There were no significant differences in major adverse cardiac event rates between the ENC and UC arms (ENC: 11 [36.7%] vs UC: 13 [32.5%]; *P* = .85; total: 24 [34.2%]) and subsequent mortality (ENC: 13 [14.8%] vs 17 [18.7%]; *P* = .62; total: 30 [16.8%]). Patients on the hospital ward randomized to the UC arm (n = 38) were more likely to be readmitted to a hospital 90 days after enrollment compared with the ENC arm (UC: 24 [63.2%] vs ENC: 15 [37.5%]; *P* = .04; total: 39 [50.0%]) (eTables 6 and 7 in Supplement 2).

**Table 3.** 90-Day Outcomes of Enrolled Patients Overall and Grouped by Treatment Arm^a^

90-d Outcome	Overall (N = 180)	ENC (n = 89)	UC (n = 91)	*P* value^b^
SCr at 90 d, median (IQR), mg/dL	1.05 (0.70-1.40)	1.10 (0.75-1.30)	1.00 (0.70-1.50)	.83
New KRT	5 (2.8)	2 (2.3)	3 (3.3)	>.99
Readmitted to the hospital	70 (39.3)	30 (34.1)	40 (44.4)	.21
Nephrology issues during readmission	22 (31.4)	10 (33.3)	12 (30.0)	.97
Major adverse cardiac issue during readmission	24 (34.2)	11 (36.7)	13 (32.5)	.85
Mortality	30 (16.8)	13 (14.8)	17 (18.7)	.62

Abbreviations: ENC, early nephrology consultation; KRT, kidney replacement therapy; SCr, serum creatinine; UC, usual care.

SI conversion factor: To convert SCr to µmol/L, multiply by 88.4.

^a^ Data are reported as No. (%) unless otherwise indicated.

^b^ Categorical variables are compared using the χ^2^ test and summarized as No. (%); continuous variables are compared by the Wilcoxon rank sum test and summarized by medians (IQRs).

### Consultation Findings and Recommendations

Across the first 7 days of enrollment, in the ENC arm, there were 121 consultations; 86 were performed on the day of enrollment in 89 patients. The other 35 consultations in the ENC arm were performed across 19 patients either on the next day to follow up on issues from the original consultation (n = 22) or to address worsening issues on day 2 or later (n = 13). In the UC arm, there were 19 unstructured consultations across 7 patients, who had a KDIGO score of 1 or more or electrolyte issues. eTable 8 in Supplement 2 describes the date and timing of all consultations across both study arms. eTable 9 in Supplement 2 describes the clinical sources and contributing factors to AKI risk and AKI according to the nephrology consultation documentation. Multiple factors and sources were present in each individual patient; volume depletion was more commonly thought to be the source of the AKI risk and early AKI in 58% (70 of 121) of the ENC consultations compared with only 11% (2 of 19) of the UC consultations.

Table 4 reports the specific recommendations that were made in the 121 ENC and 19 UC consultations and whether they were classified as completely followed (complete), partially followed (partial), or not followed at all (no). There were 270 recommendations in all of the ENC consultations and 36 in the UC consultations, among which 148 of the 306 recommendations (48%) were complete. The most common recommendations were about changing patients’ diet (ENC: 78 [28.9%]; UC: 5 [13.9%]), stopping medications (ENC: 56 [20.7%]; UC: 6 [16.7%]), and changing medication doses (ENC: 32 [11.9%]; UC: 4 [11.1%]). Recommendations around medications (dosing and stopping), diuretics or fluids, and vasopressors were completely followed in 15 of 22 UC patients (68% of time), while only 48 of 116 (41%) in the ENC cohort followed the recommendations completely.

**Table 4.** Consultant Recommendations and Inpatient Care Teams’ Adherence Classifications^a^

Consultant recommendation	Adherence classification in the ENC arm				Adherence classification in the UC arm			
	Total No.	No	Partial	Complete	Total No.	No	Partial	Complete
Medication stopping	56	30 (54)	7 (12)	19 (34)	6	1 (17)	0	5 (83)
Medication dosing	32	19 (59)	3 (9)	10 (31)	4	1 (25)	0	3 (75)
Diuretic or IVF	15	3 (20)	3 (20)	9 (60)	11	4 (36)	1 (9)	6 (55)
Vasopressors	13	3 (23)	0	10 (77)	1	0	0	1 (100)
Diet and nutrition	78	36 (46)	5 (7)	36 (46)	5	2 (40)	2 (40)	1 (100)
Potassium	12	6 (50)	0	6 (50)	1	1 (100)	0	0
Sodium	23	7 (30)	3 (13)	13 (57)	4	2 (50)	0	2 (50)
Phosphorus	26	9 (35)	1 (4)	16 (61)	3	3 (100)	0	0
Calcium	8	3 (38)	0	5 (62)	1	1 (100)	0	0
Magnesium	7	2 (28)	0	5 (72)	0	0	0	0
Total	270	118 (44)	22 (8)	130 (48)	36	15 (42)	3 (8)	18 (50)
Nondiet and nonelectrolyte^b^	116	55 (47)	13 (11)	48 (41)	22	6 (27)	1 (5)	15 (68)

Abbreviations: ENC, early nephrology consultation; IVF, intravenous fluid; UC, usual care.

^a^ Data are reported as No. (%) except for total No. Adherence classifications were completely followed (complete), partially followed (partial), or not followed at all (no).

^b^ The nondiet and nonelectrolyte category included the following categories 4 categories: (1) medication stopping, (2) medication dosing, (3) diuretic or IVF, and (4) vasopressors.

We conducted a post hoc analysis, excluding the 6 patients who developed SCr-based AKI within the 6 hours between their enrollment and randomization and the time permitted to complete the nephrology consultation (5 in the ENC arm and 1 in the UC arm) (eTables 10-12 in Supplement 2). We conducted similar analyses with the cohorts stratified by the enrollment ESTOP score (moderate risk and high risk) (eTables 13-16 in Supplement 2) and with patients enrolled before and after the spring 2020 COVID-19 pandemic enrollment pause (eTables 17-20 in Supplement 2). There were no significant changes in the outcomes in these planned (risk score) and post hoc (pandemic) analyses.

## Discussion

In this single center prospective RCT evaluating the use of a real-time machine-learning AKI risk score, we identified and enrolled a cohort of patients at high risk, of whom 38.9% developed KDIGO AKI. However, using this score to trigger an ENC, we found no statistical differences in the peak rise in SCr or the subsequent development of KDIGO AKI in those receiving an ENC. The intervention did not demonstrate a significant effect on secondary end points such as AKI severity, hospital length of stay, inpatient mortality, or 90-day outcomes. Per the study protocol, patients in the ENC arm received more consultation recommendations compared with the UC arm; however, adherence to these recommendations was low. It is possible that this low uptake of early consultative recommendations contributed to the trial outcome.

To our knowledge, our RCT is the first to use a machine-learning risk score to identify patients at high risk and then trigger an AKI-focused intervention. While other RCTs have used risk scores to implement AKI-focused intervention, these have not been machine-learning–based scores, and these investigations were done in patients after the development of KDIGO AKI.^7,11^ Other studies have targeted patients at risk for AKI but outside of the RCT setting. For example, Hodgson et al^8^ performed a controlled before and after trial, in which they enrolled both patients with established early KDIGO AKI as well as those at high risk for AKI, but they used a traditional risk score calculated at 1 time point. Thus, our trial is novel for the implementation of a cutting-edge, real-time machine-learning risk assessment tool and for targeting patients who are at risk to develop SCr-based AKI in an RCT setting.

Our findings mirror other studies investigating ENC and similar interventions in the setting of established AKI. Aklilu et al^11^ conducted an RCT in 4003 hospitalized patients with established stage 1 AKI, with approximately half of the cohort receiving personalized kidney action team recommendations directly reported to the primary team in an electronic medical record compared with UC. Only 33.8% of the kidney action team recommendations were followed (similar to our rate), and patients receiving kidney action team recommendations did not have decreased AKI progression, a need for KRT, or inpatient mortality.^11^ Similar results were reported in other trials that attempted to implement AKI care bundles in the setting of established AKI. In 2 separate trials from the UK, both Selby et al^7^ and Hodgson et al^8^ demonstrated that guideline-based care-bundle recommendations did not significantly decrease AKI progression or the need for KRT. Despite low adherence the bundle and recommendation completion (Selby et al^7^: 40.2% and Hodgson et al^8^: 19.2%), there was a signal for potentially shorter lengths of stay. While these trials differ from ours, in that we excluded patients with established AKI, our rate of adherence to recommendations was similarly under 50% (ie, 48%).

More recently, Zarbock et al^19^ completed an international RCT, in which 1180 postoperative patients at risk for AKI, as determined by an elevated urinary biomarker, were randomized to receive UC or a KDIGO care bundle (Biomarker-Guided Intervention to Prevent Acute Kidney Injury [BigpAK-2]). While they demonstrated that this care bundle decreased the incidence of stage 2 or 3 AKI (14.4% vs 22.3%), the adherence to the KDIGO bundle was 47% in the intervention arm and only 5% in the UC arm. This RCT, like ours, targeted patients prior to the presence of KDIGO AKI and strongly supports the importance of guideline-based care in preventing AKI. While it is possible that early recommendations, like in our ENC arm, are viewed as less urgent or may not be completed due to competing priorities, it is clear that implementation of guideline-based care improves patient outcomes.^18,19^ In our study, if consultation recommendations were not followed, or new issues evolved, another consultation could be written the next day to provide continued guidance. In the ENC arm, 19 of 89 patients (21%) had more than 1 consultation over the first 7 days; however, despite this vigilant care, our adherence rates remained low but also in line with rates reported in the AKI literature.^7,8,19^

While older nonrandomized data from prior to 2018 supported improved outcomes in the setting of early consultation,^20,21^ our trial adds to more recent RCT data that demonstrate limited benefit. This is perhaps because the clinical recommendations for patients in the absence of clinical SCr-based AKI were less likely to be followed. Alternatively, it is possible that evidence-based consultation recommendations may be ineffective in patients prior to the development of KDIGO AKI. It is not clear if stopping exposure to a nephrotoxin or dose reducing a medication before there is evidence of SCr-based AKI improves outcomes. Many hospitalized patients are at risk for AKI, and it may be difficult for the primary teams to determine which recommendations must be followed and which ones are less crucial. We hypothesized that those suggestions around electrolyte correction as well as diet and nutrition changes were less likely to be followed because those are issues that primary teams often manage independently of nephrology. However, the existing AKI literature demonstrates that nonadherence to care guidelines in the absence of AKI is also common.^22,23^ In an observational study, Küllmar and coauthors^22^ demonstrated that in patients at high risk for AKI after cardiac surgery, the adherence to a prespecified KDIGO care bundle was low. They demonstrated that, on average, patients and practitioners were adherent to 3.4 of 6.0 bundle measures, and bundle adherence was not significantly different between those who did and did not develop AKI. However, in the BigpAK-2 trial, early avoidance of nephrotoxins and implementation of functional hemodynamic monitoring were separately both associated with a decreased odds of developing stage 2 or 3 AKI in the first 72 hours.^19^ Future trials should investigate how many components of a care bundle need to be completed to decrease AKI and improve patient outcomes, as well as confirm which components should be prioritized in patients at high risk to optimize patient outcomes.

### Strengths and Limitations

Our study has several strengths. This is, to our knowledge, one of the first uses of a real-time machine-learning AKI risk score to trigger a clinical nephrology intervention. Our risk score, which was developed and validated in a multicenter study, was successfully implemented in the electronic medical record, and then patients were randomized to a proactive consultation intervention.^12,13^ Our risk score identified patients at risk for AKI, with no patient having AKI at enrollment, and over 1 in 3 patients developed AKI over the next 7 days. The incidence of KDIGO stage 2 or higher AKI in our enrolled population was 16.1%, which was slightly higher than the published positive predicted value of 0.01 of 12.0%. At the higher cutoff (≥0.057), the positive predicted value in the trial was 25%, slightly lower than the 27% from our development and validation cohort; thus, the score performed as expected (Table 1 and eTable 16 in Supplement 2).^12^ Additionally, 7.8% of the cohort died during their index hospitalization, and even more were readmitted (39.3%) and died (16.8%) during their 90-day follow-up. Additionally, the consultations that were highly structured to optimize data extraction and clinical understanding were done in person (face-to-face, not electronic). All consultation notes (from both study arms) were reviewed by multiple members of the research team to link their recommendations to those domains in the ENC consultations. It was not feasible to blind the inpatient care team to the consultation recommendations. However, the identification of ENC patients as high risk would bias the inpatient care team to improve care and in theory decrease the risk of developing AKI, but we did not see evidence of this. We also used standardized consensus definitions of AKI and other well-defined clinical outcomes.^5^ Thus, while our intervention failed to improve AKI-based patient outcomes, the score identified a high-risk cohort, and perhaps future studies should focus on ensuring more consultation-based recommendations are followed.

Our study also has limitations. First, it was conducted at a single academic center, and as such, the generalizability of the results is limited, and these results may not reflect community-based practices. Additionally, enrollment took longer than expected due to the COVID-19 pandemic and subsequent funding and staffing issues. We enrolled the first 32 patients in 2019 and 2020 and the remaining 148 patients in 2022 through 2024. We did not enroll any patients who tested positive for SARS-CoV-2. Since AKI was extremely common in patients with COVID-19, it is possible that the general care of all hospitalized patients with AKI changed over the course of our trial, but in our post hoc analyses, there was no difference in outcomes.^24,25^ However, we used the same machine-learning risk score during the entire trial so that all patients who we enrolled had a similar risk of severe AKI based on our prepandemic model development. Enrolled patients had a median risk score of 0.01, which we had anticipated would lead to an incidence of KDIGO stage 2 or higher AKI and an event rate of 12.0%, and we had a rate of 16.1%.^13^ Thus the risk score performed as expected.

Our negative results should not be interpreted as a failure of machine-learning risk scores but rather that valuable tools such as ESTOP should be embedded within interventions that reliably change clinical management. Additionally, despite using all available SCr from the last 6 months to establish a baseline SCr, we retrospectively identified 22 patients as having had AKI on admission based on the significant decrease (≥0.3 mg/dL or 50%) of their SCr over the study period, despite defining their baseline SCr per our protocol. This finding speaks to the limitations around establishing a baseline SCr for hospitalized patients. This underrecognized AKI, by both the research teams screening patients for enrollment and the inpatient care teams, may be prevented by only enrolling patients who have clearly established preadmission SCr, but this would limit generalizability. In addition, our findings are limited given the lack of blinding of individuals who performed the data extraction from the ENC and UC consultation notes.

## Conclusions

In this RCT, ESTOP-AKI identified a high-risk cohort of patients; however, an ENC in this cohort did not result in a decreased risk of AKI and other adverse events. Implementation of recommendations was lower in the ENC arm. Future work should investigate whether increasing adherence to early AKI care recommendations could improve clinical outcomes for patients at high risk of AKI.

## Notes

Supplement 1.
Trial Protocol

Supplement 2. **eTable 1.** The Structured Nephrology Consult **eTable 2.** Baseline Demographics and Characteristics of All Enrolled Patients (ITT) in the ICU Overall and Grouped by Treatment Arm: ENC (Early Nephrology Consult) and UC (Usual Care) **eTable 3.** Baseline Demographics and Characteristics of All Enrolled Patients (ITT) in Ward Overall and Grouped by Treatment Arm: ENC (Early Nephrology Consult) and UC (Usual Care) **eTable 4.** Inpatient Outcomes of All Enrolled Patients (ITT) in ICU Overall and Grouped by Treatment Arm: ENC (Early Nephrology Consult) and UC (Usual Care) **eTable 5.** Inpatient Outcomes of Enrolled Patients in Ward/Floor Overall and Grouped by Treatment Arm: ENC (Early Nephrology Consult) and UC (Usual Care) **eTable 6.** The 90-Day Outcomes of ICU Patients Enrolled and Grouped by Treatment Arm: ENC (Early Nephrology Consult) and UC (Usual Care) **eTable 7**. The 90-Day Outcomes of Ward Patients Enrolled and Grouped by Treatment Arm: ENC (Early Nephrology Consult) and UC (Usual Care) **eTable 8.** The Dates of Consultation and AKI Staging Across the ENC and UC Arms **eTable 9.** Potential Sources and Contributing Factors to AKI Risk and AKI per the Nephrology Consults **eTable 10.** Baseline Demographics and Characteristics of All Enrolled Patients Overall and Grouped by Treatment Arm: ENC (Early Nephrology Consult) and UC (Usual Care) (Post Hoc Removal of Those Who Developed AKI Within 6 Hours of Enrollment) **eTable 11.** Outcomes of Patients Grouped by Treatment Arm: ENC (Early Nephrology Consult) and UC (Usual Care) (Post Hoc Removal of Those Who Developed AKI Within 6 Hours of Enrollment) **eTable 12.** The 90-Day Outcomes of Enrolled Patients (Post Hoc Removal of Those Who Developed AKI Within 6 Hours of Enrollment) **eTable 13.** Baseline Demographics and Characteristics of All Enrolled Patients Overall and Grouped by Treatment Arm: ENC (Early Nephrology Consult) and UC (Usual Care) Enrolled With a Moderate ESTOP Risk Score (0.01-0.0569) **eTable 14.** Baseline Demographics and Characteristics of All Enrolled Patients Overall and Grouped by Treatment Arm: ENC (Early Nephrology Consult) and UC (Usual Care) Enrolled With a High ESTOP Risk Score (≥0.057) **eTable 15.** Outcomes of Patients Grouped by Treatment Arm: ENC (Early Nephrology Consult) and UC (Usual Care) Enrolled With a Moderate ESTOP Risk Score (0.01-0.0569) **eTable 16.** Outcomes of Patients Grouped by Treatment Arm: ENC (Early Nephrology Consult) and UC (Usual Care) Enrolled With a High ESTOP Risk Score (≥0.057) **eTable 17.** Baseline Demographics and Characteristics of All Enrolled Patients Overall and Grouped by Treatment Arm: ENC (Early Nephrology Consult) and UC (Usual Care) Enrolled Before to the Spring 2020 COVID-19 Pandemic–Based Pause in Enrollment **eTable 18.** Baseline Demographics and Characteristics of All Enrolled Patients Overall and Grouped by Treatment Arm: ENC (Early Nephrology Consult) and UC (Usual Care) Enrolled After the Spring 2020 COVID-19 Pandemic–Based Pause in Enrollment **eTable 19.** Outcomes of Patients Grouped by Treatment Arm: ENC (Early Nephrology Consult) and UC (Usual Care) Enrolled Prior to the Spring 2020 COVID-19 Pandemic Enrollment Pause **eTable 20.** Outcomes of Patients Grouped by Treatment Arm: ENC (Early Nephrology Consult) and UC (Usual Care) Enrolled After the Spring 2020 COVID-19 Pandemic Enrollment Pause **eFigure.** Serum Creatinine Over Time—Average Preadmission Baseline SCr (Base) as Well as the Enrollment (Enroll) and Subsequent 7-Day of SCr for Patients in the ENC and UC Study Arms

Supplement 3.
Nonauthor Collaborators

Supplement 4.
Data Sharing Statement
