# Supplementary material for: Early Nephrology Consultation and Acute Kidney Injury in Hospitalized Patients: A Randomized Clinical Trial
Source: JAMA Netw Open. 2026 Jul 10;9(7):e2622554. doi: 10.1001/jamanetworkopen.2026.22554 (PMC13355147; doi:10.1001/jamanetworkopen.2026.22554)
Supplement: Supplement 1. — Trial Protocol [file jamanetwopen-e2622554-s001.pdf]

PROTOCOL TITLE: An Early Real-Time Electronic Health Record Risk Algorithm for the Prevention and Treatment of Acute Kidney Injury: A Randomized Trial of an Early Standardized, Personalized Nephrology Intervention

PI: Jay Koyner, MD
773-702-3630
jkoyner@medicine.bsd.uchicago.edu

CO-I(S): James J. Dignam, PhD
Mihai Giurcanu, PhD

BACKGROUND AND RATIONALE:

AKI is a common complication in hospitalized patients and is associated with increased risk of hospital readmission, morbidity, mortality and a high cost of care[8-11]. The estimated annual cost of AKI care in the United States is over 10 billion dollars and with all-cause incidence of AKI continuing to rise, the total cost expenditure around AKI continues to increase.[10-12] Over this same decade, a consensus definition of AKI[13], new functional and biochemical biomarkers of renal injury[14,15] and advancements in electronic health records (EHR) AKI risk models have informed and advanced AKI research.[16,17] While several of these individual entities have demonstrated promise, most of this work has focused solely on critically ill ICU patients. Despite these advancements, clinicians do not have a systematic tool to reliably determine which patients will develop AKI or which will progress to more severe forms of AKI.

Over the last decade, our investigative group has worked to detect patients at highest risk for the most severe forms of AKI and the most adverse outcomes.[18-23] Using a multi-center cohort we derived and validated an EHR-based AKI risk assessment model to predict the development of creatinine based AKI *in hospital ward patients* using patient vital signs, labs and demographics (called Electronic Signal to Prevent AKI; ESTOP-AKI).[1] ESTOP-AKI, which is entirely derived from data freely available in the EHR, accurately predicts the future development of stage 3 AKI a median (IQR) of 35 (14-97) hours before any evidence of serum creatinine (SCr)-based AKI with an AUC of 0.83.[1] The University of Chicago (UofC) developed and currently utilizes a pre-existing platform to provide real-time reporting of ESTOP-AKI, as part of a real-time risk assessment scores program for ward-patients at high risk for sepsis, ICU transfer and inpatient cardiac arrest (Electronic Cardiac Arrest Risk Triage (ECART)).[24-28] Although others have developed AKI algorithms, these investigators have focused on the presences of Stage 1 AKI and thus implementation did not result in improved outcomes.[16,29,30] More recently, others have reported similar risk prediction algorithms to predict impending AKI however it remains unclear if these models can be used to improved outcomes.[31,32] ESTOP-AKI performs on par with these models, accurately predicting Stage 3 AKI 1.5 days earlier than SCr.

AKI is a diverse and complex clinical syndrome, often multifactorial in nature and no single therapeutic intervention is likely to improve outcomes. As such, we propose the intervention be a standardized early nephrology consult (ENC). ENC for AKI has been shown to associate with improved patient outcomes, lower peak SCr, less severe AKI, shorter length of hospital stay (LOS), increased renal recovery post-AKI and reduced morbidity /mortality.[3-6] In the setting of early AKI, those patients whose physicians complete all components of an AKI care bundle have better outcomes compared to those with partially completed bundles.[7] However these data arise from non-randomized trials. These same studies demonstrate that the average nephrology consultation is called 2 days after there is clinical evidence of AKI (rise in SCr or oliguria) and that 35-50% of AKI patients do not see a nephrologist until 5 days after clinical AKI. [3,5] Data from the UofC wards (Winter 2017) demonstrate the average AKI consult (n=59) occurs on hospital day 4 after a 1.6 mg/dL increase in SCr from baseline, with 44% of patients having AKI ≥Stage 2 AKI.

Specific Aim –To perform a prospective randomized clinical trial utilizing the ESTOP- AKI risk assessment model prior to measurable loss of kidney function ("pre-AKI") to determine if potential benefit (defined as lower peak SCr, lower AKI stage, shorter LOS and less morbidity and mortality) of ENC exists as compared to usual care. High-risk patients identified through ESTOP-AKI (score ≥0.0.01) will be randomized within 12 hours of consent, to receive a standardized ENC from a UofC nephrology fellow and research protocol specific attending or usual care (SOC). The standardized nephrology consultation will address pre-AKI differential diagnosis, nephrotoxins/drug dosing, volume status/renal perfusion. These specific elements were chosen as the focus of the early intervention based on prior successful AKI care bundles/ guidelines. (Table 3) [2,7,13,33] We hypothesize that high risk subjects receiving early-individualized nephrology-directed pre-AKI care will have improved outcomes (e.g. lower peak SCr, lower AKI stage, shorter LOS, fewer ICU stays and improved post-discharge renal function) compared to those receiving usual care.

Hypothesis: We hypothesize that combining the precision medicine approach of ESTOP AKI with early structured real-time individualized nephrology-centered care will improve patient outcomes (as measured by lower peak SCr, less severe AKI staging, fewer ICU transfers, shorter LOS and less mortality).

Objectives: The objective of this proposal is to transform the medical care of hospitalized patients at risk for the development of acute kidney injury (AKI) through the utilization of an automated, real-time, electronic medical record risk assessment score allowing for early standardized nephrology focused intervention.

ESTOP-AKI DEVICE

The electronic risk prediction algorithm (ESTOP-AKI) being studied will interface with Epic to determine the likelihood for the patient to develop AKI. The study team is creating the algorithm with the help of software engineers and architects from UofC CBIS. ESTOP-AKI will use the following information from Epic:

- Medical Record Number
- Name
- Age
- Sex
- vital signs (respiratory rate, heart rate, temperature, pulse pressure index, systolic and diastolic blood pressure, oxygen saturation, and mental status)
- laboratory values (complete blood count, liver function panel, and blood chemistries/ basic metabolic panel).

Upon generation of a risk level, ESTOP-AKI will electronically notify a member of the study team the eligibility status of the patient.

STUDY DESIGN

Type of Study: Single-center Prospective Randomized Interventional Trial

Duration of Study: 3 years

Schedule of Events

Event	Screening	Enrollment (Day 0)	Daily (Days 1-7)	Hospital Discharge	Follow Up (Month 3, 6,9, and 12)
Demographics	X				
Inclusion/Exclusion	X				
ESTOP-AKI Risk Assessment	X				
Informed Consent		X			
Blood Collection[c]		X	X	X	
Urine Collection[c]		X	X	X	
Early Nephrology Consult		X[a]			
Chart Review[b]	X[b]	X[b]	X[b]	X[b]	X[b]
Follow Up Phone Calls					X

Primary Endpoint Definition -The primary endpoint of interest, Δ-SCr, is the peak change from study entry in SCr level over a 7-day interval. We define Δ-SCr as the maximal change in creatinine over this interval, and aim to detect a clinical difference in Δ-SCr between the SOC and ENC treatment groups. On average, in patients with an ESTOP-AKI score ≥0.01 (the enrollment cutoff), the mean change in SCr in a 7-day interval was 0.369, (standard deviation 0.734).

Secondary Endpoint: Secondary endpoints include differences by treatment group in the proportion and time to event of patients who a) develop > Stage 2 AKI, b) require RRT, c) undergo ICU transfer, and d) require non-research nephrology consult. The LOS and the distributions of time to peak SCr will also be compared.

SUBJECT SELECTION AND WITHDRAWAL

Number of Subjects: 180 patients admitted to UofC Hospital over a 24 month period (90 per arm)

Inclusion Criteria

a) Age >18 years old
b) Initial E-STOP AKI score ≥0.01 within the last 12 hours.

Exclusion Criteria

a) Voluntary refusal or missing written consent of the patient / legal representative.
b) Patients with a known history of end-stage renal disease on dialysis (including renal transplantation).
c) Patients without a measured serum creatinine value during their inpatient stay.
d) Patients with a creatinine >4.0 mg/dl at the time of admission or available in the EHR from the last 6 months [16]
e) Patients with prior episode of KDIGO defined AKI during this same hospitalization- regardless of E-STOP AKI score
f) Patients with prior renal consultation during their admission.

Location: All research activities will take place at the University of Chicago Medical Center. Data will not be shared outside the institution or with investigators outside the study team. De-identified

data may be placed into NIH databases, such as dbGaP, as this is a NIH-funded study. Security measures are in place to protect these data

Informed Consent Process The investigator will make certain that an appropriate informed consent process is in place to ensure that potential research subjects, or their authorized representatives, are fully informed about the nature and objectives of the clinical study, the potential risks and benefits of study participation, and their rights as research subjects. The investigator, or a sub-investigator(s) designated by the sponsor-investigator, will obtain the written, signed informed consent of each subject, or prior to performing any study-specific procedures on the subject. The date and time that the subject signs the informed consent form and a narrative of the issues discussed during the informed consent process will be documented in the subject's case history. The investigator will retain the original copy of the signed informed consent form, and a copy will be provided to the subject.

The investigator will make certain that appropriate processes and procedures are in place to ensure that ongoing questions and concerns of enrolled subjects are adequately addressed and that the subjects are informed of any new information that may affect their decision to continue participation in the clinical study. In the event of substantial changes to the clinical study or the risk-to-benefit ratio of study participation, the sponsor-investigator will obtain the informed consent of enrolled subjects for continued participation in the clinical study.

STUDY PROCEDURES

Screening: As part of ESTOP-AKI production, the algorithm which assesses risk on a real time basis will be reported in EPIC but not labeled as an AKI risk score . Dr. Koyner's research team will approach eligible patients (and their treating physicians) about study enrollment, utilizing a UofC model in place from prior work by Dr. Koyner and David Meltzer's. These pre-emptive and consistent in-servicing of treating physicians/nurses, research staff being omnipresent with dedicated ward-based work space and establishing a dedicated pager/contact numbers for the study with 24 hour coverage.

Randomization: Once consented patients will be randomized to ENC or usual care through an on-line RED-CAP based block-randomization schema.

Study Visits: Following enrollment, subjects will receive a research ENC on Day 0 and the consultation recommendations will be discussed with the primary team and will be placed in the EHR within 8 hours of enrollment. The research team will be made up of a rotating panel of UofC nephrology attendings specifically trained for this protocol Following hospital discharge, there will be a 3 month (±2 weeks) follow up phone call.

Intervention: The ENC will provide guidance on Differential Diagnosis, Nephrotoxins/Drug Dosing and Volume Status/ Renal Perfusion). The research ENC will have daily follow up with the inpatient team and document recommendations based on these 3 core elements. At any time during the study any patient in either arm can receive a formal non-research based renal consultation at the discretion of the treating physicians and these events will be captured. Once a non-research based consult has been called the patient will no longer be seen by the research team but will still be eligible for data collection, blood and urine and follow up testing. As we anticipate some proportion of patients in both arms will receive non-research consultations we will be tracking how many patients in receive nephrology consultation as well as other diagnostic aspects of AKI care in order to determine the degree of contamination in the usual care arm (see below). **No patient will be denied a non-research based consult at any time**.

Blood, and urine collection: We will collect up to 10 ml of blood and 20ml of urine from each recipient at daily for the first 7days. Samples will be promptly centrifuged, aliquoted and will be stored at -80°C as previously described.[20,21,23,84]Samples will be used to measure SCr and electrolytes should they not be measured clinically on a given day. Excess samples will be bio-banked for future biomarker (biochemical and genetic) investigation to determine if novel tests may guide clinical care in patients at high risk for severe AKI per E-STOP-AKI. Any shared samples would be de-identified prior to transfer; no clinical data will accompany the samples.

De-identified biobanked samples will be shared with external collaborators, including Sphingotec GmbH, for advanced biomarker analysis. These analyses will focus on the evaluation of proenkephalin and other novel biomarkers to enhance the prediction and management of AKI.

Data to be collected: We will systematically retrieve pre-determined variables for each participant, including demographics (age, gender, race), comorbidities (reason for hospitalization, AKI risk factor, severity of illness, diabetes, CHF, COPD, hypertension, pre-enrollment renal function and medication profile), operative variables (e.g. cardiopulmonary bypass time, transfusions, hypotension), hospital prescribed medications (vaso-active medications, ACE-I, ARBs, nephrotoxins). Vital signs, fluid balance and urine output for days 1-7 will be recorded. Data will be entered into a pre-established online secure password protected REDCAP database. Data integrity will be checked with 5% random accuracy models.[20,23,84]

STATISTICAL PLAN AND CONSIDERATIONS

Sample Size Determination To detect a mean difference in Δ-creatinine of ≥0.35 mg/dL at conventional two-sided (type I error) $\alpha = 0.05$ and power of 80% requires measurements on 65 patients per treatment group (130 total). **This** sample size of 65 patients per group does not provide adequate power around the secondary endpoint to detect a decrease in the development of stage 2 AKI. At this size we have 80% power to detect a 70.9% reduction the development of Stage 2 AKI. At a cutoff of greater than 0.01 over 30% of all patients enrolled are at risk for developing Stage 2 AKI. Thus by enrolling 90 patients per arm we are able to ensure that we achieve 80% power for the primary and have 80% power to detect a 60% reduction in the development of Stage 2 AKI. Should every patient enrolled stay in the study we will have even more power approaching 90% power for the primary endpoint.

Statistical Considerations: *Randomization* Patients will be randomized 1:1 to two arms (standard of care (SOC) vs. ENC using an ESTOP-AKI stratified REDCAP-based algorithm. Assignments will be made within strata of ESTOP-AKI risk scores (>0.01 to 0.569 and ≥0.57 to protect against imbalances in randomization on key factors (age, race, baseline SCr, severity of illness, patient location). To balance the number assigned per arm, permuted assignment blocks of variable size that are unknown to investigators will be used.

Analytic Methods - ***Primary endpoint:*** Difference of mean Δ-SCr between the ENC and SOC group. Randomized assignment stratified by predicted risk (ESTOP-AKI score) maximizes the potential for equal distribution of potential confounding factors by treatment group. Primary analysis is thus a comparison of means from a 2-factor (treatment and stratification level) ANOVA model. If there is evidence of imbalance in key factors (baseline SCr, age, sex**,** hospital service type**),** then a secondary analysis using linear model methods will be conducted to compare adjusted means. For patients who die within 7-days or receive RRT we will analyze the data with Δ-SCr set at their maximum SCr prior these events. ***Secondary endpoints:*** For binary outcomes (i.e., AKI stage, ICU transfer), proportions will be compared between ENC and SOC groups via the binomial test. The primary analysis will be a direct comparison of these proportions. Logistic regression analysis may be conducted to account for covariate imbalances. Time to discharge (LOS) and time to peak SCr distributions will be compared by group using nonparametric statistics for differences in specific

quantiles (e.g. medians) and other summaries (means). These endpoints can also be approached from a survival analysis modeling perspective, with treatment and other covariates investigated in relation to event rates.

RISKS AND BENEFITS

Risks The only risk to subjects is minimal risk associated with blood draw which include bruising and irritation at the sight of penetration. Also subjects are at minimal risk for loss of confidentiality.

Benefits to subject: There may or may not be direct medical benefit to those subjects included in the intervention group.

Interim Monitoring – An independent Data and Safety Monitoring Board (DSMB) and its membership has been established for this trial. Study conduct and progress will be reviewed on multiple occasions with a mandatory analysis once 20-25% enrollment (~ 20 per arm) for safety and feasibility as well as a futility / efficacy analysis at 50% enrollment (~40 per arm).

REFERENCES

1. Koyner JL, Adhikari R, Edelson DP, Churpek MM. Development of a Multicenter Ward-Based AKI Prediction Model. *Clin J Am Soc Nephrol.* 2016;11(11):1935-1943.
2. Kolhe NV, Staples D, Reilly T, et al. Impact of Compliance with a Care Bundle on Acute Kidney Injury Outcomes: A Prospective Observational Study. *PloS one.* 2015;10(7):e0132279.
3. Balasubramanian G, Al-Aly Z, Moiz A, et al. Early nephrologist involvement in hospital-acquired acute kidney injury: a pilot study. *Am J Kidney Dis.* 2011;57(2):228-234.
4. Costa e Silva VT, Liano F, Muriel A, Diez R, de Castro I, Yu L. Nephrology referral and outcomes in critically ill acute kidney injury patients. *PloS one.* 2013;8(8):e70482.
5. Meier P, Bonfils RM, Vogt B, Burnand B, Burnier M. Referral patterns and outcomes in noncritically ill patients with hospital-acquired acute kidney injury. *Clin J Am Soc Nephrol.* 2011;6(9):2215-2225.
6. Flores-Gama C, Merino M, Baranda F, Cruz DN, Ronco C, Vazquez-Rangel A. The impact of integrating nephrologists into the postoperative cardiac intensive care unit: a cohort study. *Cardiorenal medicine.* 2013;3(1):79-88.
7. Kolhe NV, Reilly T, Leung J, et al. A simple care bundle for use in acute kidney injury: a propensity score matched cohort study. *Nephrol Dial Transplant.* 2016.
8. Brown JR, Parikh CR, Ross CS, et al. Impact of perioperative acute kidney injury as a severity index for thirty-day readmission after cardiac surgery. *Ann Thorac Surg.* 2014;97(1):111-117.
9. Chertow G, Levy E, Hammermeister K, Grover F, Daley J. Independent association between acute renal failure and mortality following caridac surgery. *Am J Med.* 1998;104:343-348.
10. Hobson C, Ozrazgat-Baslanti T, Kuxhausen A, et al. Cost and Mortality Associated With Postoperative Acute Kidney Injury. *Annals of surgery.* 2015;261(6):1207-1214.
11. Chertow GM, Burdick E, Honour M, Bonventre JV, Bates DW. Acute kidney injury, mortality, length of stay, and costs in hospitalized patients. *J Am Soc Nephrol.* 2005;16(11):3365-3370.

12. Hsu RK, McCulloch CE, Dudley RA, Lo LJ, Hsu CY. Temporal changes in incidence of dialysis-requiring AKI. *J Am Soc Nephrol.* 2013;24(1):37-42.
13. KDIGO. Kidney Disease: Improving Global Outcomes (KDIGO) Acute Kidney Injury Work Group. KDIGO Clinical Practice Guideline for Acute Kidney Injury. *Kidney Int.* 2012;Supp(2):1-138.
14. Chen LX, Koyner JL. Biomarkers in Acute Kidney Injury. *Critical care clinics.* 2015;31(4):633-648.
15. Koyner JL, Parikh CR. Clinical Utility of Biomarkers of AKI in Cardiac Surgery and Critical Illness. *Clin J Am Soc Nephrol.* 2013;8(6):1034-1042.
16. Wilson FP, Shashaty M, Testani J, et al. Automated, electronic alerts for acute kidney injury: a single-blind, parallel-group, randomised controlled trial. *Lancet.* 2015;385(9981):1966-1974.
17. Kashani K, Herasevich V. Utilities of Electronic Medical Records to Improve Quality of Care for Acute Kidney Injury: Past, Present, Future. *Nephron.* 2015;131(2):92-96.
18. Chawla LS, Davison DL, Brasha-Mitchell E, et al. Development and Standardization of a Furosemide Stress Test to Predict the Severity of Acute Kidney Injury. *Crit Care.* 2013;17(5):R207.
19. Kashani K, Al-Khafaji A, Ardiles T, et al. Discovery and validation of cell cycle arrest biomarkers in human acute kidney injury. *Crit Care.* 2013;17(1):R25.
20. Koyner JL, Davison DL, Brasha-Mitchell E, et al. Furosemide Stress Test and Biomarkers for the Prediction of AKI Severity. *J Am Soc Nephrol.* 2015.
21. Koyner JL, Garg AX, Coca SG, et al. Biomarkers predict progression of acute kidney injury after cardiac surgery. *J Am Soc Nephrol.* 2012;23(5):905-914.
22. Koyner JL, Shaw AD, Chawla LS, et al. Tissue Inhibitor Metalloproteinase-2 (TIMP-2)IGF-Binding Protein-7 (IGFBP7) Levels Are Associated with Adverse Long-Term Outcomes in Patients with AKI. *J Am Soc Nephrol.* 2015;26(7):1747-1754.
23. Koyner JL, Vaidya VS, Bennett MR, et al. Urinary biomarkers in the clinical prognosis and early detection of acute kidney injury. *Clin J Am Soc Nephrol.* 2010;5(12):2154-2165.
24. Churpek MM, Adhikari R, Edelson DP. The value of vital sign trends for detecting clinical deterioration on the wards. *Resuscitation.* 2016;102:1-5.
25. Churpek MM, Yuen TC, Park SY, Gibbons R, Edelson DP. Using electronic health record data to develop and validate a prediction model for adverse outcomes in the wards*. *Crit Care Med.* 2014;42(4):841-848.
26. Churpek MM, Yuen TC, Winslow C, et al. Multicenter development and validation of a risk stratification tool for ward patients. *Am J Respir Crit Care Med.* 2014;190(6):649-655.
27. Churpek MM, Zadravecz FJ, Winslow C, Howell M, Edelson DP. Incidence and Prognostic Value of the Systemic Inflammatory Response Syndrome and Organ Dysfunctions in Ward Patients. *Am J Respir Crit Care Med.* 2015.
28. Kang MA, Churpek MM, Zadravecz FJ, Adhikari R, Twu NM, Edelson DP. Real-Time Risk Prediction on the Wards: A Feasibility Study. *Crit Care Med.* 2016.
29. Mehta R, Bihorac A, Selby NM, et al. Establishing a continuum of acute kidney injury - tracing AKI using data source linkage and long-term follow-up: Workgroup Statements from the 15th ADQI Consensus Conference. *Canadian journal of kidney health and disease.* 2016;3:13.

30. Selby NM, Crowley L, Fluck RJ, et al. Use of electronic results reporting to diagnose and monitor AKI in hospitalized patients. *Clin J Am Soc Nephrol.* 2012;7(4):533-540.
31. Saly D, Yang A, Triebwasser C, et al. Approaches to Predicting Outcomes in Patients with Acute Kidney Injury. *PloS one.* 2017;12(1):e0169305.
32. Flechet M, Guiza F, Schetz M, et al. AKIpredictor, an online prognostic calculator for acute kidney injury in adult critically ill patients: development, validation and comparison to serum neutrophil gelatinase-associated lipocalin. *Intensive Care Med.* 2017.
33. Meersch M, Schmidt C, Hoffmeier A, et al. Prevention of cardiac surgery-associated AKI by implementing the KDIGO guidelines in high risk patients identified by biomarkers: the PrevAKI randomized controlled trial. *Intensive Care Med.* 2017.
34. Bagshaw SM, Goldstein SL, Ronco C, Kellum JA. Acute kidney injury in the era of big data: the 15(th) Consensus Conference of the Acute Dialysis Quality Initiative (ADQI). *Canadian journal of kidney health and disease.* 2016;3:5.
35. Hoste EA, Kashani K, Gibney N, et al. Impact of electronic-alerting of acute kidney injury: workgroup statements from the 15(th) ADQI Consensus Conference. *Canadian journal of kidney health and disease.* 2016;3:10.
36. James MT, Hobson CE, Darmon M, et al. Applications for detection of acute kidney injury using electronic medical records and clinical information systems: workgroup statements from the 15(th) ADQI Consensus Conference. *Canadian journal of kidney health and disease.* 2016;3:9.
37. Siew ED, Basu RK, Wunsch H, et al. Optimizing administrative datasets to examine acute kidney injury in the era of big data: workgroup statement from the 15(th) ADQI Consensus Conference. *Canadian journal of kidney health and disease.* 2016;3:12.
38. Sutherland SM, Chawla LS, Kane-Gill SL, et al. Utilizing electronic health records to predict acute kidney injury risk and outcomes: workgroup statements from the 15(th) ADQI Consensus Conference. *Canadian journal of kidney health and disease.* 2016;3:11.
39. Ahmed A, Vairavan S, Akhoundi A, et al. Development and validation of electronic surveillance tool for acute kidney injury: A retrospective analysis. *Journal of critical care.* 2015.
40. Kashani K, Herasevich V. Sniffing out acute kidney injury in the ICU: do we have the tools? *Curr Opin Crit Care.* 2013;19(6):531-536.
41. Xu X, Nie S, Liu Z, et al. Epidemiology and Clinical Correlates of AKI in Chinese Hospitalized Adults. *Clin J Am Soc Nephrol.* 2015;10(9):1510-1518.
42. Bellomo R, Kellum JA, Ronco C. Acute kidney injury. *Lancet.* 2012;380(9843):756-766.
43. Koyner JL, Cerda J, Goldstein SL, et al. The daily burden of acute kidney injury: a survey of U.S. nephrologists on World Kidney Day. *Am J Kidney Dis.* 2014;64(3):394-401.
44. Cerda J, Lameire N, Eggers P, et al. Epidemiology of acute kidney injury. *Clin J Am Soc Nephrol.* 2008;3(3):881-886.
45. Susantitaphong P, Cruz DN, Cerda J, et al. World Incidence of AKI: A Meta-Analysis. *Clin J Am Soc Nephrol.* 2013.
46. Lenihan CR, Montez-Rath ME, Mora Mangano CT, Chertow GM, Winkelmayer WC. Trends in acute kidney injury, associated use of dialysis, and mortality after cardiac surgery, 1999 to 2008. *Ann Thorac Surg.* 2013;95(1):20-28.

47. Desai AA, Baras J, Berk BB, et al. Management of acute kidney injury in the intensive care unit: a cost-effectiveness analysis of daily vs alternate-day hemodialysis. *Arch Intern Med.* 2008;168(16):1761-1767.
48. Parikh A, Shaw A. The economics of renal failure and kidney disease in critically ill patients. *Critical care clinics.* 2012;28(1):99-111, vii.
49. Chawla LS, Eggers PW, Star RA, Kimmel PL. Acute kidney injury and chronic kidney disease as interconnected syndromes. *N Engl J Med.* 2014;371(1):58-66.
50. Siew ED, Parr SK, Abdel-Kader K, et al. Predictors of Recurrent AKI. *J Am Soc Nephrol.* 2016;27(4):1190-1200.
51. Kiers HD, van den Boogaard M, Schoenmakers MC, et al. Comparison and clinical suitability of eight prediction models for cardiac surgery-related acute kidney injury. *Nephrol Dial Transplant.* 2013;28(2):345-351.
52. Chertow GM, Lazarus JM, Christiansen CL, et al. Preoperative renal risk stratification. *Circulation.* 1997;95(4):878-884.
53. Mehta RH, Grab JD, O'Brien SM, et al. Bedside tool for predicting the risk of postoperative dialysis in patients undergoing cardiac surgery. *Circulation.* 2006;114(21):2208-2216; quiz 2208.
54. Thakar CV, Arrigain S, Worley S, Yared JP, Paganini EP. A clinical score to predict acute renal failure after cardiac surgery. *J Am Soc Nephrol.* 2005;16(1):162-168.
55. Palomba H, de Castro I, Neto AL, Lage S, Yu L. Acute kidney injury prediction following elective cardiac surgery: AKICS Score. *Kidney Int.* 2007;72(5):624-631.
56. Wijeysundera DN, Karkouti K, Dupuis JY, et al. Derivation and validation of a simplified predictive index for renal replacement therapy after cardiac surgery. *Jama.* 2007;297(16):1801-1809.
57. Aronson S, Fontes ML, Miao Y, Mangano DT. Risk index for perioperative renal dysfunction/failure: critical dependence on pulse pressure hypertension. *Circulation.* 2007;115(6):733-742.
58. Rahmanian PB, Kwiecien G, Langebartels G, Madershahian N, Wittwer T, Wahlers T. Logistic risk model predicting postoperative renal failure requiring dialysis in cardiac surgery patients. *Eur J Cardiothorac Surg.* 2011;40(3):701-707.
59. Fortescue EB, Bates DW, Chertow GM. Predicting acute renal failure after coronary bypass surgery: cross-validation of two risk-stratification algorithms. *Kidney Int.* 2000;57(6):2594-2602.
60. Englberger L, Suri RM, Li Z, et al. Validation of clinical scores predicting severe acute kidney injury after cardiac surgery. *Am J Kidney Dis.* 2010;56(4):623-631.
61. Mehta RL, Pascual MT, Gruta CG, Zhuang S, Chertow GM. Refining predictive models in critically ill patients with acute renal failure. *J Am Soc Nephrol.* 2002;13(5):1350-1357.
62. Huen SC, Parikh CR. Predicting acute kidney injury after cardiac surgery: a systematic review. *Ann Thorac Surg.* 2012;93(1):337-347.
63. Brown JR, Cochran RP, Leavitt BJ, et al. Multivariable prediction of renal insufficiency developing after cardiac surgery. *Circulation.* 2007;116(11 Suppl):I139-143.
64. Bullock ML, Umen AJ, Finkelstein M, Keane WF. The assessment of risk factors in 462 patients with acute renal failure. *Am J Kidney Dis.* 1985;5(2):97-103.
65. Lohr JW, McFarlane MJ, Grantham JJ. A clinical index to predict survival in acute renal failure patients requiring dialysis. *Am J Kidney Dis.* 1988;11(3):254-259.

66. Liano F, Gallego A, Pascual J, et al. Prognosis of acute tubular necrosis: an extended prospectively contrasted study. *Nephron.* 1993;63(1):21-31.
67. Chertow GM, Levy EM, Hammermeister KE, Grover F, Daley J. Independent association between acute renal failure and mortality following cardiac surgery. *Am J Med.* 1998;104(4):343-348.
68. Chertow GM, Soroko SH, Paganini EP, et al. Mortality after acute renal failure: models for prognostic stratification and risk adjustment. *Kidney Int.* 2006;70(6):1120-1126.
69. Paganini EP, Halstenberg WK, Goormastic M. Risk modeling in acute renal failure requiring dialysis: the introduction of a new model. *Clin Nephrol.* 1996;46(3):206-211.
70. Lins RL, Elseviers MM, Daelemans R, et al. Re-evaluation and modification of the Stuivenberg Hospital Acute Renal Failure (SHARF) scoring system for the prognosis of acute renal failure: an independent multicentre, prospective study. *Nephrol Dial Transplant.* 2004;19(9):2282-2288.
71. Demirjian S, Chertow GM, Zhang JH, et al. Model to predict mortality in critically ill adults with acute kidney injury. *Clin J Am Soc Nephrol.* 2011;6(9):2114-2120.
72. Basu RK, Wang Y, Wong HR, Chawla LS, Wheeler DS, Goldstein SL. Incorporation of biomarkers with the renal angina index for prediction of severe AKI in critically ill children. *Clin J Am Soc Nephrol.* 2014;9(4):654-662.
73. Basu RK, Zappitelli M, Brunner L, et al. Derivation and validation of the renal angina index to improve the prediction of acute kidney injury in critically ill children. *Kidney Int.* 2014;85(3):659-667.
74. Gaudry S, Ricard JD, Leclaire C, et al. Acute kidney injury in critical care: experience of a conservative strategy. *Journal of critical care.* 2014;29(6):1022-1027.
75. Sawyer AM, Deal EN, Labelle AJ, et al. Implementation of a real-time computerized sepsis alert in nonintensive care unit patients. *Crit Care Med.* 2011;39(3):469-473.
76. Escobar GJ, Ragins A, Scheirer P, Liu V, Robles J, Kipnis P. Nonelective Rehospitalizations and Postdischarge Mortality: Predictive Models Suitable for Use in Real Time. *Medical care.* 2015;53(11):916-923.
77. Siew ED, Peterson JF, Eden SK, et al. Outpatient nephrology referral rates after acute kidney injury. *J Am Soc Nephrol.* 2012;23(2):305-312.
78. Ponce D, Zorzenon Cde P, dos Santos NY, Balbi AL. Early nephrology consultation can have an impact on outcome of acute kidney injury patients. *Nephrol Dial Transplant.* 2011;26(10):3202-3206.
79. Faubel S, Chawla LS, Chertow GM, et al. Ongoing clinical trials in AKI. *Clin J Am Soc Nephrol.* 2012;7(5):861-873.
80. Bellomo R, Cass A, Cole L, et al. Intensity of continuous renal-replacement therapy in critically ill patients. *N Engl J Med.* 2009;361(17):1627-1638.
81. Palevsky PM, Zhang JH, O'Connor TZ, et al. Intensity of renal support in critically ill patients with acute kidney injury. *N Engl J Med.* 2008;359(1):7-20.
82. Heung M, Koyner JL. Entanglement of sepsis, chronic kidney disease, and other comorbidities in patients who develop acute kidney injury. *Seminars in nephrology.* 2015;35(1):23-37.
83. Koyner J, Shaw A, Chawla LS, et al. Increased TIMP2*IGFBP7 is associated with Increased 9 Month Mortality in ICU Patients at Risk for AKI. *J Am Soc Nephrol.* 2013;24:40A.

84. Koyner JL, Bennett MR, Worcester EM, et al. Urinary cystatin C as an early biomarker of acute kidney injury following adult cardiothoracic surgery. *Kidney Int.* 2008;23:23.
85. Koyner JL, Cerda J, Goldstein SL, et al. The Daily Burden of Acute Kidney Injury: A World Kidney Day Survey of U.S. Nephrologists. *Journal of the American Society of Nephrology.* 2013;24(Oral Abstract Presented at ASN Renal Week - Thursday November 7, 2013):3A.
86. Koyner JL, Garg AX, Coca SG, et al. Biomarkers Predict Progression of Acute Kidney Injury after Cardiac Surgery. *J Am Soc Nephrol.* 2012.
87. Koyner JL, Garg AX, Shlipak MG, et al. Urinary cystatin C and acute kidney injury after cardiac surgery. *Am J Kidney Dis.* 2013;61(5):730-738.
88. Parikh CR, Coca SG, Thiessen-Philbrook H, et al. Postoperative Biomarkers Predict Acute Kidney Injury and Poor Outcomes after Adult Cardiac Surgery. *J Am Soc Nephrol.* 2011;22(9):1748-1757.
89. Parikh CR, Devarajan P, Zappitelli M, et al. Postoperative Biomarkers Predict Acute Kidney Injury and Poor Outcomes after Pediatric Cardiac Surgery. *J Am Soc Nephrol.* 2011;22(9):1737-1747.
90. Parikh CR, Thiessen-Philbrook H, Garg AX, et al. Performance of Kidney Injury Molecule-1 and Liver Fatty Acid-Binding Protein and Combined Biomarkers of AKI after Cardiac Surgery. *Clin J Am Soc Nephrol.* 2013.
91. Coca SG, Garg AX, Thiessen-Philbrook H, et al. Urinary Biomarkers of AKI and Mortality 3 Years after Cardiac Surgery. *J Am Soc Nephrol.* 2013.
92. Go AS, Parikh CR, Ikizler TA, et al. The assessment, serial evaluation, and subsequent sequelae of acute kidney injury (ASSESS-AKI) study: design and methods. *BMC Nephrol.* 2010;11:22.
