# Supplementary material for: Early Nephrology Consultation and Acute Kidney Injury in Hospitalized Patients: A Randomized Clinical Trial
Source: JAMA Netw Open. 2026 Jul 10;9(7):e2622554. doi: 10.1001/jamanetworkopen.2026.22554 (PMC13355147; doi:10.1001/jamanetworkopen.2026.22554)
Supplement: Supplement 2. — eTable 1. The Structured Nephrology Consult eTable 2. Baseline Demographics and Characteristics of All Enrolled Patients (ITT) in the ICU Overall and Grouped by Treatment Arm: ENC (Early Nephrology Consult) and UC (Usual Care) eTable 3. Baseline Demographics and Characteristics of All Enrolled Patients (ITT) in Ward Overall and Grouped by Treatment Arm: ENC (Early Nephrology Consult) and UC (Usual Care) eTable 4. Inpatient Outcomes of All Enrolled Patients (ITT) in ICU Overall and Grouped by Treatment Arm: ENC (Early Nephrology Consult) and UC (Usual Care) eTable 5. Inpatient Outcomes of Enrolled Patients in Ward/Floor Overall and Grouped by Treatment Arm: ENC (Early Nephrology Consult) and UC (Usual Care) eTable 6. The 90-Day Outcomes of ICU Patients Enrolled and Grouped by Treatment Arm: ENC (Early Nephrology Consult) and UC (Usual Care) eTable 7. The 90-Day Outcomes of Ward Patients Enrolled and Grouped by Treatment Arm: ENC (Early Nephrology Consult) and UC (Usual Care) eTable 8. The Dates of Consultation and AKI Staging Across the ENC and UC Arms eTable 9. Potential Sources and Contributing Factors to AKI Risk and AKI per the Nephrology Consults eTable 10. Baseline Demographics and Characteristics of All Enrolled Patients Overall and Grouped by Treatment Arm: ENC (Early Nephrology Consult) and UC (Usual Care) (Post Hoc Removal of Those Who Developed AKI Within 6 Hours of Enrollment) eTable 11. Outcomes of Patients Grouped by Treatment Arm: ENC (Early Nephrology Consult) and UC (Usual Care) (Post Hoc Removal of Those Who Developed AKI Within 6 Hours of Enrollment) eTable 12. The 90-Day Outcomes of Enrolled Patients (Post Hoc Removal of Those Who Developed AKI Within 6 Hours of Enrollment) eTable 13. Baseline Demographics and Characteristics of All Enrolled Patients Overall and Grouped by Treatment Arm: ENC (Early Nephrology Consult) and UC (Usual Care) Enrolled With a Moderate ESTOP Risk Score (0.01-0.0569) eTable 14. Baseline Demographics and Characteristics of [file jamanetwopen-e2622554-s002.pdf]

Supplemental Online Content

Churpek MM, Fatima A, Anjorin O, et al. Early nephrology consultation and acute kidney injury in hospitalized patients at risk: a randomized clinical trial. *JAMA Netw Open*. 2026;9(7):e2622554. doi:10.1001/jamanetworkopen.2026.22554

eTable 1. The Structured Nephrology Consult
eTable 2. Baseline Demographics and Characteristics of All Enrolled Patients (ITT) in the ICU Overall and Grouped by Treatment Arm: ENC (Early Nephrology Consult) and UC (Usual Care)
eTable 3. Baseline Demographics and Characteristics of All Enrolled Patients (ITT) in Ward Overall and Grouped by Treatment Arm: ENC (Early Nephrology Consult) and UC (Usual Care)
eTable 4. Inpatient Outcomes of All Enrolled Patients (ITT) in ICU Overall and Grouped by Treatment Arm: ENC (Early Nephrology Consult) and UC (Usual Care)
eTable 5. Inpatient Outcomes of Enrolled Patients in Ward/Floor Overall and Grouped by Treatment Arm: ENC (Early Nephrology Consult) and UC (Usual Care)
eTable 6. The 90-Day Outcomes of ICU Patients Enrolled and Grouped by Treatment Arm: ENC (Early Nephrology Consult) and UC (Usual Care)
eTable 7. The 90-Day Outcomes of Ward Patients Enrolled and Grouped by Treatment Arm: ENC (Early Nephrology Consult) and UC (Usual Care)
eTable 8. The Dates of Consultation and AKI Staging Across the ENC and UC Arms
eTable 9. Potential Sources and Contributing Factors to AKI Risk and AKI per the Nephrology Consults
eTable 10. Baseline Demographics and Characteristics of All Enrolled Patients Overall and Grouped by Treatment Arm: ENC (Early Nephrology Consult) and UC (Usual Care) (Post Hoc Removal of Those Who Developed AKI Within 6 Hours of Enrollment)
eTable 11. Outcomes of Patients Grouped by Treatment Arm: ENC (Early Nephrology Consult) and UC (Usual Care) (Post Hoc Removal of Those Who Developed AKI Within 6 Hours of Enrollment)
eTable 12. The 90-Day Outcomes of Enrolled Patients (Post Hoc Removal of Those Who Developed AKI Within 6 Hours of Enrollment)
eTable 13. Baseline Demographics and Characteristics of All Enrolled Patients Overall and Grouped by Treatment Arm: ENC (Early Nephrology Consult) and UC (Usual Care) Enrolled With a Moderate ESTOP Risk Score (0.01-0.0569)
eTable 14. Baseline Demographics and Characteristics of All Enrolled Patients Overall and Grouped by Treatment Arm: ENC (Early Nephrology Consult) and UC (Usual Care) Enrolled With a High ESTOP Risk Score (≥0.057)
eTable 15. Outcomes of Patients Grouped by Treatment Arm: ENC (Early Nephrology Consult) and UC (Usual Care) Enrolled With a Moderate ESTOP Risk Score (0.01-0.0569)
eTable 16. Outcomes of Patients Grouped by Treatment Arm: ENC (Early Nephrology Consult) and UC (Usual Care) Enrolled With a High ESTOP Risk Score (≥0.057)
eTable 17. Baseline Demographics and Characteristics of All Enrolled Patients Overall and Grouped by Treatment Arm: ENC (Early Nephrology Consult) and UC (Usual Care) Enrolled Before to the Spring 2020 COVID-19 Pandemic–Based Pause in Enrollment

© 2026 Churpek MM et al. *JAMA Network Open*.

eTable 18. Baseline Demographics and Characteristics of All Enrolled Patients Overall and Grouped by Treatment Arm: ENC (Early Nephrology Consult) and UC (Usual Care) Enrolled After the Spring 2020 COVID-19 Pandemic–Based Pause in Enrollment
eTable 19. Outcomes of Patients Grouped by Treatment Arm: ENC (Early Nephrology Consult) and UC (Usual Care) Enrolled Prior to the Spring 2020 COVID-19 Pandemic Enrollment Pause
eTable 20. Outcomes of Patients Grouped by Treatment Arm: ENC (Early Nephrology Consult) and UC (Usual Care) Enrolled After the Spring 2020 COVID-19 Pandemic Enrollment Pause
eFigure. Serum Creatinine Over Time—Average Preadmission Baseline SCr (Base) as Well as the Enrollment (Enroll) and Subsequent 7-Day of SCr for Patients in the ENC and UC Study Arms

This supplemental material has been provided by the authors to give readers additional information about their work.

© 2026 Churpek MM et al. *JAMA Network Open*.

eTable 1. The Structured Nephrology Consult

Volume Status

I think the patient is (Volume Status) – choices Hypovolemic, Euvolemic, Hypervolemic

In order to get the patient to an improved volume status the patient should
(Fluid or Volume Administration) choices
Do Nothing
Receive Packed Red Blood Cells (If yes, how many)
Receive IV albumin (if yes how much and how frequent)
Receive IV Crystalloid (if yes what type and how much

- 0.9% Saline
- 0.45% Saline
- Lactated Ringers
- D5W with 3 amps of Bicarbonate
- Other

Receive Diuretics (if yes what type, what route and how frequent)

- Furosemide (Lasix) IV/PO
- Bumetanide (Bumex) IV/PO
- HCTZ IV/PO
- Metolazone (Zaroxlyn)
- Spironolactone
- Other

Renal Perfusion
Is there a role for increasing the renal perfusion? Yes/No (if no skip this section)
If yes, please consider using one of the following vaso-active agents to help improve renal perfusion...

- Norepinephrine
- Vasopressin
- Epinephrine
- Phenylephrine
- Angiotensin II
- Dopamine
- Dobutamine
- Milrinone
- Othe

Medication Selection / Dosing
The patient is on the following medication which potentially require dose adjustment / changes.

- Metformin
- Vancomycin
- Cephalosporin
- Penicillin
- Antiviral
- Allopurinol
- Other

© 2026 Churpek MM et al. *JAMA Network Open*.

Please consider changing the dosing regimen of these medications and / or consulting your team's pharmacist.

Nutritional Needs
1) Please work to ensure that the patient maintains a normal blood sugar level, as hyperglycemia has been repeatedly been shown to increase the risk of severe AKI and AKI progression.
2) If the patient is on an oral diet please consider placing the patient on a (Diet options choices)

- Renal Diet
- Regular Diet
- Heart Healthy- 2 gram Sodium Diet

3) If the patient is on tube feeds, please consider changing them to a low potassium and low phosphorus feed, like Nephro, although protein needs and other factors need to be considered.

Electrolytes
Sodium - are there issues / concerns with the patient's sodium? (Yes/ No)
A) The sodium is elevated and the patient needs free water (how much over what time course)
B) The sodium is low, please consider the following: (Low sodium option)

- Volume expansion with IV Fluids (How Much and Type)
- Fluid Restriction (volume)
- Salt Tabs
- ADH-based therapy

Potassium - are there issues / concerns with the patient's potassium? (Yes/ No)

A) The potassium is elevated - please consider (High potassium options – can choose more than one)
   - Change in medications (e.g. Renin-Aldosterone agents, Heparin, Trimethoprim…)
   - Start on a new medication (Potassium binders)
   - Change in diet / tube feeds (see nutrition section)
B) The potassium is low - please consider (Low potassium option can choose more than one)
   - Repletion with IV or PO potassium (how much / how often)
   - Stopping or changing a medication (diuretics, drugs causing stool losses)

Phosphorus - are there issues / concerns with the patient's phosphorus (Yes/ No)

A) The phosphorus is elevated and the patient needs to (High phos options can choose more than one)
   - Start on a new medication (e.g. calcium acetate, sevelamer carbonate etc..)
   - Change in diet or tube feeds (see nutrition section)
   - Dialysis / Renal Replacement Therapy

B) The phosphorus is low, your patient need needs to have their phosphorus supplemented with either PO or IV Phos. Please give them (how much and how often)

Calcium - are there issues or concerns with the patient's Calcium (Yes/ No)

A) The Calcium is elevated, and the patient needs: (High Calcium Options can choose more than one)
   - IV fluids (non-hyperchloremic in concert with loop diuretics (how much and how often)
   - Calcitonin

© 2026 Churpek MM et al. *JAMA Network Open*.

- Bisphosphonates
- Dialysis / Renal Replacement Therapy
- Other (steroids, denosumab)

B) The calcium is low, your patient need needs to have their calcium supplemented with either PO or IV calcium. Please give them (how much and timing)

Magnesium - are there issues or concerns with the patient's Magnesium (Yes/ No)

A) The Magnesium is elevated, and the patient needs (High Magnesium Options – can choose more than one)
   - Diuretics (how much and how frequent)
   - Dialysis / Renal Replacement Therapy
   - Other (steroids, denosumab)

B) The Magnesium is low, your patient needs (Low Magnesium Options can choose more than one)
   - Repletion with IV or PO Magnesium (how much how frequent)
   - Stopping or changing a medication (e.g. diuretics)
   - Other

Further testing (urine studies, follow up blood work, imaging etc....

Foley - Does the patient require an indwelling bladder catheter? (Foley Catheter Options)

- No they already have one
- No they do not need one
- Yes, please place a catheter

Imaging - Does the patient require any form of renal imaging? (Yes/No)
If Yes, please order:

- Renal Ultrasound
- CT Scan without IV contrast
- Other

Urine Studies : Please consider sending the following urine studies on the patient:

- Urinalysis
- Urine Sodium
- Urine Urea
- Urine Creatinine
- Urine Osmolarity
- Urine Culture
- NephroCheck (AKI-Risk)
- Other urine tests

Blood Works / Studies: Please consider sending the following studies on the patient:
(Blood Work ESTOP)

- Repeat Basic Metabolic Panel, Magnesium, Phosphate

© 2026 Churpek MM et al. *JAMA Network Open*.

- Uric Acid
- Cystatin C
- Creatine Kinase
- Blood Cultures
- Complement studies
- Other

© 2026 Churpek MM et al. *JAMA Network Open*.

eTable 2. Baseline Demographics and Characteristics of All Enrolled Patients (ITT) in the ICU Overall and Grouped by Treatment Arm: ENC (Early Nephrology Consult) and UC (Usual Care)

Categorical variables are compared using the chi-squared test and summarized as counts and percentages; continuous variables are compared by rank-sum test and summarized by medians and interquartile range.

		Overall	ENC	UC
N		101	48	53
Age		64.0 [52.0, 72.0]	60.5 [50.75, 71.0]	66.0 [55.0, 75.0]
Gender	Male	62 (61.4)	32 (66.7)	30 (56.6)
Race	White	56 (55.4)	22 (52.1)	31 (58.5)
	Black	40 (39.6)	21 (43.8)	19 (35.8)
	Other	5 (5.0)	2 (4.2)	3 (5.7)
Hispanic		4 (4.0)	3 (6.2)	1 (1.9)
Past Medical History				
Hypertension		57 (56.4)	26 (54.2)	31 (58.5)
Diabetes		28 (27.7)	17 (35.4)	11 (20.8)
Chronic Kidney Disease		8(7.9)	2(4.2)	6 (11.3)
Congestive Heart Failure		11 (10.9)	4 (8.3)	7 (13.2)
Cancer		19 (18.8)	10 (20.8)	9 (17.0)
ESTOP at Enrollment		0.01 [0.01, 0.02]	0.01 [0.01, 0.02]	0.01 [0.01, 0.01]
Max ESTOP Prior to Enrollment		0.02 [0.01, 0.04]	0.02 [0.01, 0.03]	0.02 [0.01, 0.04]
ESTOP Risk Stratification Group	High risk (≥0.057)	13 (12.9)	7 (14.6)	6 (11.3)
	Moderate risk (0.01-0.0569)	88 (87.1)	41 (85.4)	47 (88.7)
Time from Admission to Enrollment (hours)		42.4 [41.4 – 65.6]	42.3 [41.1-65.0]	43.0 [41.5 – 89.9]
Mechanical Ventilation at Enrollment		32 (31.7)	17 (35.4)	15 (28.3)
Foley Catheter at Enrollment		67 (66.3)	29 (60.4)	38 (71.7)
SOFA at Enrollment		4.00 [3.00, 6.25]	3.50 [2.75, 5.25]	4.00 [3.00, 7.00]
APACHE II at Enrollment		15.00 [12.00, 21.00]	15.00 [12.00, 20.00]	17.50 [14.25, 21.00]
Pre-Hospitalization Baseline SCr		0.99 (0.42)	0.93 (0.35)	1.04(0.48)
Enrollment SCr		1.03 (0.38)	0.99(0.35)	1.07(0.41)
Enrollment Blood Urea Nitrogen		24.2 (17.3)	23.6 (17.1)	24.7 (17.6)

© 2026 Churpek MM et al. *JAMA Network Open*.

Race and Hispanic ethnicity were reported as recorded by the electronic health record. Other race includes – Asian and Other. All values reported as either No(%) or median IQR

© 2026 Churpek MM et al. *JAMA Network Open*.

eTable 3. Baseline Demographics and Characteristics of All Enrolled Patients (ITT) in Ward Overall and Grouped by Treatment Arm: ENC (Early Nephrology Consult) and UC (Usual Care)

Categorical variables are compared using the chi-squared test and summarized as counts and percentages; continuous variables are compared by the rank-sum test and summarized by medians and interquartile range.

		Overall	ENC	UC
n		79	41	38
Age		60.0 [48.5, 70.0]	57.0 [48.0, 67.0]	63.0 [50.0, 71.0]
Gender	Male	40 (50.6)	21 (51.2)	19 (50.0)
Race	White	39 (49.4)	18 (43.9)	21 (55.3)
	Black	34 (43.0)	17 (41.5)	16 (44.7)
	Other	6 (7.7)	6 (14.6)	0 (0.0)
Hispanic		2 (2.6)	1 (2.4)	1 (2.6)
Past Medical History				
Hypertension		47 (59.5)	24 (58.5)	23 (60.5)
Diabetes		19 (24.1)	12 (29.3)	7 (18.4)
Chronic Kidney disease		4 (5.1)	3 (7.3)	1 (2.6)
Congestive Heart Failure		12 (15.2)	8 (19.5)	4 (10.5)
Cancer		27 (34.2)	12 (29.3)	15 (39.5)
ESTOP at Enrollment		0.01 [0.01, 0.02]	0.01 [0.01, 0.01]	0.01 [0.01, 0.03]
Max ESTOP Prior to Enrollment		0.02 [0.01, 0.03]	0.01 [0.01, 0.02]	0.02 [0.01, 0.03]
ESTOP Risk Stratification Group	High risk (≥0.057)	3 (3.8)	2 (4.9)	1 (2.6)
	Moderate risk (0.01-0.0569)	76 (96.2)	39 (95.1)	37 (97.4)
Time from Admission to Enrollment (hours)		89.1 [64.5 – 163.2]	68.1 [43.3-138.0]	90.0 [65.0 – 187.0]
Foley Catheter at Enrollment		21 (26.6)	8 (19.5)	13 (34.2)
MEWS at Enrollment		3.00 [2.00, 4.00]	3.00 [2.00, 4.00]	3.00 [2.00, 4.75]
Pre-Hospitalization Baseline SCr		1.05(0.45)	1.03 (0.37)	1.08 (0.53)
Enrollment SCr		1.11 (0.48)	1.08 (0.40)	1.14 (0.55)
Enrollment Blood Urea Nitrogen		21.4 (15.9)	22.0(13.7)	20.7 (18.2)
Race and Hispanic ethnicity were reported as recorded by the electronic health record. Other race includes – Asian and Other. All values reported as either No(%), Mean (SD) or median IQR				

© 2026 Churpek MM et al. *JAMA Network Open*.

eTable 4. Inpatient Outcomes of All Enrolled Patients (ITT) in ICU Overall and Grouped by Treatment Arm: ENC (Early Nephrology Consult) and UC (Usual Care)

Categorical variables are compared using the chi-squared test and summarized as counts and percentages; continuous variables are compared by the rank-sum test and summarized by medians and interquartile range.

		Overall	EC	UC	P-value
n		101	48	53	
Peak SCr during first 7 days post enrollment mean (SD)		1.07(0.71)	1.08 (0.73)	1.07 (0.70)	0.95
Peak Change in SCr from enrollment mean (SD)		0.03 (0.54)	0.08 (0.58)	-0.02 (0.49)	0.37
Dialysis (%)		4 (4.0)	2 (4.2)	2 (3.8)	>0.99
Death (%)		12 (11,9)	6 (12.5)	6 (11.3)	>0.99
Maximum KDIGO Stage					0.29
	No AKI	51 (50.5)	20 (41.7)	31 (58.5)	
	One	26 (25.7)	13 (27.1)	13 (24.5)	
	Two	19 (18.8)	12 (25.0)	7 (13.2)	
	Three	5 (5.0)	3 (6.2)	2 (3.8)	
Hospital Length of Stay (Days)		8.00 [5.00, 14.00]	9.00 [5.75, 13.50]	8.00 [4.00, 14.00]	0.44
ICU Length of Stay (Days)		3.00 [2.00, 7.00]	4.00 [2.00, 6.50]	3.00 [1.00, 7.00]	0.50
SCr – Serum Creatinine (mg/dL) All values reported as either No(%), mean(SD) or median IQR					

© 2026 Churpek MM et al. *JAMA Network Open*.

eTable 5. Inpatient Outcomes of Enrolled Patients in Ward/Floor Overall and Grouped by Treatment Arm: ENC (Early Nephrology Consult) and UC (Usual Care)

Categorical variables are compared using the chi-squared test and summarized as counts and percentages; continuous variables are compared by rank-sum test and summarized by medians and interquartile range.

		Overall	ENC	UC	P-value
n		79	41	38	
Peak SCr during first 7 days post enrollment mean (SD)		1.10 (0.62)	1.09 (0.55)	1.10 (0.68)	0.94
Peak Change in SCr from enrollment mean (SD)		-0.01 (0.41)	0.01 (0.43)	-0.04 (0.39)	0.60
Dialysis		1 (1.3)	1 (2.4)	0 (0.0)	>0.99
Death		2 (2.5)	2 (4.9)	0 (0.0)	0.49
Maximum KDIGO Stage					0.79
	No AKI	59 (74.7)	32 (78.0)	27 (71.1)	
	One	15 (19.0)	7 (17.1)	8 (21.1)	
	Two	5 (6.3)	2 (4.9)	3 (7.9)	
	Three	0 (0.0)	0 (0.0)	0 (0.0)	
Hospital Length of Stay (Days)		10.00 [6.00, 17.00]	9.00 [6.00, 17.00]	11.50 [6.25, 19.00]	0.33
ICU Length of Stay (Days)		3.00 [2.00, 3.00]	2.50 [2.25, 2.75]	3.00 [2.50, 7.50]	0.54
SCr – Serum Creatinine (mg/dL) All values reported as either No(%) , Mean (SD) or median IQR					

© 2026 Churpek MM et al. *JAMA Network Open*.

eTable 6. The 90-Day Outcomes of ICU Patients Enrolled and Grouped by Treatment Arm: ENC (Early Nephrology Consult) and UC (Usual Care)

Categorical variables are compared using the chi-squared test and summarized as counts and percentages; continuous variables are compared by the rank-sum test and summarized by medians and interquartile range.

	Overall	ENC	UC	P-value
n	101	48	53	
SCr	1.00 [0.70, 1.42]	1.00 [0.80, 1.25]	1.00 [0.60, 1.50]	0.89
New RRT	2 (2.0)	0 (0.0)	2 (3.8)	0.50
Readmitted to the Hospital	31 (31.0)	15 (31.2)	16 (30.8)	>0.99
Nephrology Issues during Readmission	8 (25.8)	5 (33.3)	3 (18.8)	0.43
Major Adverse Cardiac Issue during Readmission	13.0 (41.9)	4 (26.7)	9 (56.3)	0.15
Mortality	19 (18.8)	7 (14.6)	12 (22.6)	0.44
SCr – serum creatinine (mg/dL) - All values reported as either No(%) or median IQR				

© 2026 Churpek MM et al. *JAMA Network Open*.

eTable 7. The 90-Day Outcomes of Ward Patients Enrolled and Grouped by Treatment Arm: ENC (Early Nephrology Consult) and UC (Usual Care)

Categorical variables are compared using the chi-squared test and summarized as counts and percentages; continuous variables are compared by the rank-sum test and summarized by medians and interquartile range.

	Overall	ENC	UC	P-value
n	79	41	38	
SCr	1.10 [0.70, 1.33]	1.10 [0.70, 1.30]	1.00 [0.70, 1.52]	0.91
New RRT	3 (3.8)	2 (5.0)	1 (2.6)	>0.99
Readmitted to the Hospital	39 (50.0)	15 (37.5)	24 (63.2)	0.041
Nephrology Issues during Readmission	14 (35.9)	5 (33.3)	9 (37.5)	>0.99
Major Adverse Cardiac Issue during Readmission	11 (28.2)	7 (46.7)	4 (16.7)	0.07
Mortality	11 (14.1)	6 (15.0)	5 (13.2)	>0.99
SCr – serum creatinine (mg/dL) - All values reported as either No(%) or median IQR				

© 2026 Churpek MM et al. *JAMA Network Open*.

eTable 8. The Dates of Consultation and AKI Staging Across the ENC and UC Arms

	Day 0-Enrollment	Day 1	Day 2	Day 3	Day 4	Day 5	Day 6	Day 7
**ENC (n=89)**								
**Number of Consults**	**86**	**11**	**9**	**4**	**1**	**1**	**4**	**5**
**No AKI**	**86**	**4**	**3**	**1**	**0**	**0**	**0**	**0**
**Stage 1**	**0**	**3**	**1**	**1**	**1**	**1**	**3**	**3**
**Stage 2**	**0**	**3**	**3**	**0**	**0**	**0**	**1**	**1**
**Stage 3**	**0**	**1**	**2**	**2**	**0**	**0**	**0**	**1**
**UC arm (n=91)**								
**Number of Consults**	**2**	**2**	**3**	**3**	**4**	**1**	**1**	**3**
**No AKI**	**2**	**2**	**0**	**1**	**1**	**0**	**0**	**0**
**Stage 1**	**0**	**0**	**2**	**1**	**2**	**1**	**0**	**1**
**Stage 2**	**0**	**0**	**1**	**1**	**1**	**0**	**0**	**1**
**Stage 3**	**0**	**0**	**0**	**0**	**0**	**0**	**1**	**1**

© 2026 Churpek MM et al. *JAMA Network Open*.

eTable 9. Potential Sources and Contributing Factors to AKI Risk and AKI per the Nephrology Consults

	Early Nephrology Consult Arm (n=121 consults in 89 patients)	Usual Care Arm (19 consults in 91 patients)
**Potential Sources of AKI n(%)**		
Volume Depletion	70 (58)	2 (11)
Nephrotoxin Associated	54 (45)	8 (42)
Cardio Renal	17 (14)	4 (21)
Cardiac Surgery	16 (13)	1 (5)
Sepsis	20 (17)	0 (0)
Obstruction	3 (2)	0 (0)
Hypertensive Urgency	1 (1)	0 (0)
**Contributing Factors to AKI n(%)**		
Poor Forward Flow /renal perfusion	37 (31)	4 (21)
Renal Congestion	12 (10)	5 (26)
Contrast Exposure	28 (23)	7 (43)
Vancomycin Exposure	12 (10)	0 (0)
Calcineurin Inhibitor Exposure	3 (2)	0(0)
Chemotherapy/ Immunotherapy Exposure	11 (0)	1(5)
**ATN diagnosis**	51 (42)	10 (53)

© 2026 Churpek MM et al. *JAMA Network Open*.

eTable 10. Baseline Demographics and Characteristics of All Enrolled Patients Overall and Grouped by Treatment Arm: ENC (Early Nephrology Consult) and UC (Usual Care) (Post Hoc Removal of Those Who Developed AKI Within 6 Hours of Enrollment)

		Overall	ENC	UC
n		174	84	90
Age		62.5 [50.0, 71.0]	59.0 [50.0, 70.0]	65.00 [53.0, 72.0]
Gender	Male	99 (56.9)	50 (59.5)	49 (54.4)
Race	White	92 (52.9)	40 (47.6)	52 (57.8)
	Black	71 (40.8)	36 (42.9)	35 (38.9)
	Asian	4 (2.3)	3 (3.6)	1 (1.1)
	Native American	1 (0.5)	1 (1.2)	0 (0.0)
	Other	6 (3.4)	4 (4.8)	2 (2.2)
Hispanic		6 (3.4)	4 (4.8)	2 (2.2)
Past Medical History				
Hypertension		99 (56.9)	46 (54.8)	53 (58.9)
Diabetes		46 (26.4)	28 (33.3)	18 (20.0)
Chronic Kidney Disease		10 (5.7)	3 (3.6)	7 (7.8)
Congestive Heart Failure		23 (13.2)	12 (14.3)	11 (12.2)
Cancer		46 (26.4)	22 (26.2)	24 (26.7)
ESTOP at Enrollment		0.01 [0.01, 0.02]	0.01 [0.01, 0.02]	0.01 [0.01, 0.02]
Max ESTOP Prior to Enrollment		0.02 [0.01, 0.03]	0.02 [0.01, 0.03]	0.02 [0.01, 0.03]
ESTOP Risk Group	High risk (≥0.057)	14 (8.0)	8 (9.5)	6 (6.7)
	Moderate risk (0.01-0.0569)	160 (92.0)	76 (90.5)	84 (93.3)
Patient Location at Randomization	Ward/Floor	78 (44.8)	41 (48.8)	37 (41.1)
	ICU	96 (55.2)	43 (51.2)	53 (58.9)
Time from Admission to Enrollment (hours)		65.0 [42.0 – 114.5]	65.1 [41.8-90.1]	65.0 [42.1 – 115.4]
Mechanical Ventilation at Enrollment		35 (19.8)	16 (18.6)	19 (20.9)
Foley Catheter at Enrollment		85 (48.0)	34 (39.5)	51 (56.0)
SOFA at Enrollment for ICU patients		4.00 [3.00, 6.00]	3.00 [2.50, 5.00]	4.00 [3.00, 7.00]
MEWS at Enrollment for Ward patients		3.00 [2.00, 4.00]	3.00 [2.00, 4.00]	3.00 [2.00, 5.00]
APACHE II at Enrollment for ICU patients		15.00 [12.00, 20.75]	14.50 [11.75, 20.00]	17.50 [14.25, 21.00]

© 2026 Churpek MM et al. *JAMA Network Open*.

Pre-Hospitalization Baseline SCr		0.90 [0.72, 1.22]	0.90 [0.72, 1.16]	0.88 [0.73, 1.30]
Enrollment SCr		1.01 [0.74, 1.25]	1.03 [0.75, 1.24]	1.00 [0.72, 1.28]
Enrollment Blood Urea Nitrogen		19.00 [12.00, 28.00]	19.00 [12.50, 28.50]	20.50 [12.00, 28.00]
Race and Hispanic ethnicity were reported as recorded by the electronic health record. Other race includes – Asian, Unknown and Mixed. All values reported as either No(%) or median IQR				

Categorical variables are compared using the chi-squared test and summarized as counts and percentages; continuous variables are compared with the rank-sum test and summarized by medians and interquartile range.

© 2026 Churpek MM et al. *JAMA Network Open*.

eTable 11. Outcomes of Patients Grouped by Treatment Arm: ENC (Early Nephrology Consult) and UC (Usual Care) (Post Hoc Removal of Those Who Developed AKI Within 6 Hours of Enrollment)

Categorical variables are compared using the chi-squared test and summarized as counts and percentages; continuous variables are compared with the rank-sum test and summarized by medians and interquartile range.

		Overall	ENC	UC	P-value
n		174	84	90	
Peak SCr during first 7 days post enrollment		1.10 [0.88, 1.47]	1.17 [0.96, 1.43]	1.06 [0.80, 1.53]	0.58
Peak Change in SCr from enrollment		0.00 [-0.08, 0.14]	0.00 [-0.08, 0.18]	0.00 [-0.07, 0.10]	0.89
Peak Change in SCr from pre-admission baseline		0.07 [-0.06, 0.26]	0.10 [-0.02, 0.27]	0.04 [-0.10, 0.22]	0.18
Dialysis		4 (2.3)	2 (2.4)	2 (2.2)	0.99
Death		14 (8.0)	8 (9.5)	6 (6.7)	0.68
Maximum KDIGO Stage	No AKI	110 (63.2)	52 (61.9)	58 (64.4)	0.86
	One	39 (22.4)	18 (21.4)	21 (23.3)	
	Two	21 (12.1)	12 (14.3)	9 (10.0)	
	Three	4 (2.3)	2 (2.4)	2 (2.2)	
Duration of All AKI (n=64)		2.0 [1.0, 3.0]	2.0 [1.0, 3.0]	2.0 [1.0, 3.75]	0.46
Duration of Severe AKI (Stage 2 or 3) n=25		2.0 [2.0, 4.0]	3.0 [2.0, 3.0]	2.0 [2.0, 6.0]	0.88
Hospital Length of Stay (Days)		9.00 [5.00, 15.75]	9.00 [5.75, 13.50]	9.00 [5.00, 17.00]	0.83
ICU Length of Stay (Days)		3.00 [2.00, 6.50]	3.00 [2.00, 6.00]	3.00 [1.25, 7.00]	0.82
SCr – serum creatinine (mg/dL) - All values reported as either No(%),mean(SD) or median IQR					

© 2026 Churpek MM et al. *JAMA Network Open*.

eTable 12. The 90-Day Outcomes of Enrolled Patients (Post Hoc Removal of Those Who Developed AKI Within 6 Hours of Enrollment)

Categorical variables are compared using the chi-squared test and summarized as counts and percentages; continuous variables are compared by the rank-sum test and summarized by medians and interquartile range.

	Overall	ENC	UC	P-value
n	174	84	90	
SCr	1.00 [0.70, 1.40]	1.10 [0.70, 1.30]	1.00 [0.67, 1.52]	0.71
New RRT	5 (2.9)	2 (2.4)	3 (3.3)	0.93
Readmitted to the Hospital	66 (37.9)	27 (32.1)	39 (43.3)	0.31
Nephrology Issues during Readmission	20 (11.5)	8 (9.5)	12 (13.3)	0.31
Major Adverse Cardiac Issue during Readmission	23 (13.2)	11 (13.1)	12 (13.3)	0.62
Mortality	30 (17.2)	13 (15.5)	17 (18.9)	0.50
SCr – serum creatinine (mg/dL) - All values reported as either No(%) or median IQR				

© 2026 Churpek MM et al. *JAMA Network Open*.

eTable 13. Baseline Demographics and Characteristics of All Enrolled Patients Overall and Grouped by Treatment Arm: ENC (Early Nephrology Consult) and UC (Usual Care) Enrolled With a Moderate ESTOP Risk Score (0.01-0.0569)

		Overall	ENC	UC
n		164	80	84
Age		62.00 [50.00, 71.00]	60.00 [50.75, 70.00]	63.50 [49.75, 72.25]
Gender	Male	89 (54.3)	46 (57.5)	43 (51.2)
Race	White	86 (52.4)	40 (50.0)	46 (54.8)
	Black	69 (42.1)	34 (42.5)	35 (41.7)
	Other	9 (5.5)	6 (7.5)	3 (3.6)
Hispanic		4 (2.4)	2 (2.5)	2 (2.4)
Past Medical History				
Hypertension		95 (57.9)	47 (58.8)	48 (57.1)
Diabetes		43 (26.2)	27 (33.8)	16 (19.0)
Chronic Kidney Disease		11 (6.7)	4 (5.0)	7 (8.3)
Congestive Heart Failure		20 (12.2)	11 (13.8)	9 (10.7)
Cancer		41 (25.0)	20 (25.0)	21 (25.0)
ESTOP at Enrollment		0.01 [0.01, 0.02]	0.01 [0.01, 0.01]	0.01 [0.01, 0.02]
Max ESTOP Prior to Enrollment		0.02 [0.01, 0.03]	0.01 [0.01, 0.02]	0.02 [0.01, 0.03]
Patient Location at Randomization	Ward/Floor	76 (46.3)	39 (48.8)	37 (44.0)
	ICU	88 (53.7)	41 (51.2)	47 (56.0)
Time from Admission to Enrollment (hours)		65.1 [42.0, 114.5]	65.2 [42.0, 90.1]	65.0 [42.1, 115.4]
Mechanical Ventilation at Enrollment		30 (18.3)	14 (17.5)	16 (19.0)
Foley Catheter at Enrollment		80 (48.8)	32 (40.0)	48 (57.1)
SOFA at Enrollment for ICU patients		4.00 [3.00, 7.00]	3.00 [3.00, 5.00]	4.00 [3.00, 7.00]
MEWS at Enrollment for Ward patients		3.00 [2.00, 4.00]	3.00 [2.00, 4.00]	3.00 [2.00, 5.00]
APACHE II at Enrollment for ICU patients		15.00 [12.00, 20.00]	14.00 [11.50, 15.00]	18.50 [15.00, 21.00]
Pre-Hospitalization Baseline SCr		1.01 (0.44)	0.96 (0.35)	1.05 (0.51)
Enrollment SCr		1.05 (0.43)	1.01 (0.36)	1.09 (0.48)
Enrollment Blood Urea Nitrogen		23.0 (17.3)	22.9 (16.3)	23.1(18.4)
Race and Hispanic ethnicity were reported as recorded by the electronic health record. Other race includes – Asian, Unknown and Mixed. All values reported as either No(%), Mean(SD) or median IQR				

© 2026 Churpek MM et al. *JAMA Network Open*.

Categorical variables are compared using the chi-squared test and summarized as counts and percentages; continuous variables are compared with the rank-sum test and summarized by medians and interquartile range.

© 2026 Churpek MM et al. *JAMA Network Open*.

eTable 14. Baseline Demographics and Characteristics of All Enrolled Patients Overall and Grouped by Treatment Arm: ENC (Early Nephrology Consult) and UC (Usual Care) Enrolled With a High ESTOP Risk Score (≥0.057)

		Overall	ENC	UC
n		16	9	7
Age		65.00 [52.25, 69.50]	53.00 [47.00, 61.00]	69.00 [68.00, 73.00]
Gender	Male	13 (81.2)	7 (77.8)	6 (85.7)
Race	White	9 (56.2)	3 (33.3)	6 (85.7)
	Black	5 (31.2)	4 (44.4)	1 (14.3)
	Other	2 (12.5)	2 (22.2)	0 (0.0)
Hispanic		2 (12.5)	2 (22.2)	0 (0.0)
Past Medical History				
Hypertension		9 (56.2)	3 (33.3)	6 (85.7)
Diabetes		4 (25.0)	2 (22.2)	2 (28.6)
Chronic Kidney Disease		1 (6.2)	1 (11.1)	0 (0.0)
Congestive Heart Failure		3 (18.8)	1 (11.1)	2 (28.6)
Cancer		5 (31.2)	2 (22.2)	3 (42.9)
ESTOP at Enrollment		0.05 [0.03, 0.06]	0.04 [0.02, 0.05]	0.06 [0.06, 0.06]
Max ESTOP Prior to Enrollment		0.07 [0.06, 0.08]	0.08 [0.07, 0.08]	0.06 [0.06, 0.07]
Patient Location at Randomization	Ward/Floor	3 (18.8)	2 (22.2)	1 (14.3)
	ICU	13 (81.2)	7 (77.8)	6 (85.7)
Time from Admission to Enrollment (hours)		42.13 [40.85, 95.71]	41.75 [41.00, 42.43]	90.00 [51.70, 125.88]
Mechanical Ventilation at Enrollment		7 (43.8)	4 (44.4)	3 (42.9)
Foley Catheter at Enrollment		8 (50.0)	5 (55.6)	3 (42.9)
SOFA at Enrollment for ICU patients		4.00 [3.00, 5.00]	4.00 [1.50, 8.00]	4.50 [3.25, 5.00]
MEWS at Enrollment for Ward patients		1.00 [1.00, 1.50]	1.50 [1.25, 1.75]	1.00 [1.00, 1.00]
APACHE II at Enrollment for ICU patients		19.50 [12.25, 26.50]	26.00 [16.00, 28.50]	13.50 [12.00, 17.25]
Pre-Hospitalization Baseline SCr		1.12 (0.40)	1.12 (0.50)	1.12 (0.28)
Enrollment SCr		1.21 (0.42)	1.21 (0.48)	1.22 (0.35)
Enrollment Blood Urea Nitrogen		22.6 (8.9)	22.6 (7.4)	22.6 (11.2)
Race and Hispanic ethnicity were reported as recorded by the electronic health record. Other race includes – Asian, Unknown and Mixed. All values reported as either No(%) mean(SD) or median IQR				

© 2026 Churpek MM et al. *JAMA Network Open*.

Categorical variables are compared using the chi-squared test and summarized as counts and percentages; continuous variables are compared with the rank-sum test and summarized by medians and interquartile range.

© 2026 Churpek MM et al. *JAMA Network Open*.

eTable 15. Outcomes of Patients Grouped by Treatment Arm: ENC (Early Nephrology Consult) and UC (Usual Care) Enrolled With a Moderate ESTOP Risk Score (0.01-0.0569)

Categorical variables are compared using the chi-squared test and summarized as counts and percentages; continuous variables are compared with the rank-sum test and summarized by medians and interquartile range.

		Overall	ENC	UC	P-value
n		164	80	84	
Peak SCr during first 7 days post enrollment		1.05 (0.43)	1.01 (0.36)	1.09 (0.48)	0.26
Peak Change in SCr from enrollment		1.21 (0.63)	1.23 (0.65)	1.20 (0.61)	0.71
Peak Change in SCr from pre-admission baseline		0.16 (0.39)	0.22 (0.44)	0.10 (0.32)	0.05
Dialysis		4 (2.4)	3 (3.8)	1 (1.2)	0.58
Death		11 (6.7)	7 (8.8)	4 (4.8)	0.48
Maximum KDIGO Stage	No AKI	101 (61.6)	46 (57.5)	55 (65.5)	0.64
	One	38 (23.2)	19 (23.8)	19 (22.6)	
	Two	20 (12.2)	12 (15.0)	8 (9.5)	
	Three	5 (3.0)	3 (3.8)	2 (2.4)	
Duration of All AKI (n=64)		13.66 (15.02)	13.78 (15.04)	13.55 (15.09)	0.92
Duration of Severe AKI (Stage 2 or 3) n=25		5.39 (5.84)	5.74 (6.04)	5.08 (5.71)	0.60
Hospital Length of Stay (Days)		1.05 (0.43)	1.01 (0.36)	1.09 (0.48)	0.26
ICU Length of Stay (Days)		1.21 (0.63)	1.23 (0.65)	1.20 (0.61)	0.71
SCr – serum creatinine (mg/dL) - All values reported as either No(%) or mean(SD)					

© 2026 Churpek MM et al. *JAMA Network Open*.

eTable 16. Outcomes of Patients Grouped by Treatment Arm: ENC (Early Nephrology Consult) and UC (Usual Care) Enrolled With a High ESTOP Risk Score (≥0.057)

Categorical variables are compared using the chi-squared test and summarized as counts and percentages; continuous variables are compared with the rank-sum test and summarized by medians and interquartile range.

		Overall	ENC	UC	P-value
n		16	9	7	
Peak SCr during first 7 days post enrollment		1.21 (0.42)	1.21 (0.48)	1.22 (0.35)	0.94
Peak Change in SCr from enrollment		1.40 (0.75)	1.31 (0.49)	1.51 (1.04)	0.6
Peak Change in SCr from pre-admission baseline		0.18 (0.50)	0.10 (0.12)	0.29 (0.76)	0.47
Dialysis		1 (6.2)	0 (0.0)	1 (14.3)	0.90
Death		3 (18.8)	1 (11.1)	2 (28.6)	0.81
Maximum KDIGO Stage	No AKI	9 (56.2)	6 (66.7)	3 (42.9)	0.58
	One	3 (18.8)	1 (11.1)	2 (28.6)	
	Two	4 (25.0)	2 (22.2)	2 (28.6)	
	Three	0 (0.0)	0 (0.0)	0 (0.0)	
Duration of All AKI (n=64)		10.31 (7.77)	8.11 (4.43)	13.14 (10.40)	0.21
Duration of Severe AKI (Stage 2 or 3) n=25		4.31 (4.17)	4.71 (5.53)	3.83 (2.14)	0.72
Hospital Length of Stay (Days)		1.21 (0.42)	1.21 (0.48)	1.22 (0.35)	0.94
ICU Length of Stay (Days)		1.40 (0.75)	1.31 (0.49)	1.51 (1.04)	0.6
SCr – serum creatinine (mg/dL) - All values reported as either No(%,) or mean(SD)					

© 2026 Churpek MM et al. *JAMA Network Open*.

eTable 17. Baseline Demographics and Characteristics of All Enrolled Patients Overall and Grouped by Treatment Arm: ENC (Early Nephrology Consult) and UC (Usual Care) Enrolled Before to the Spring 2020 COVID-19 Pandemic–Based Pause in Enrollment

		Overall	ENC	UC
n		32	15	17
Age		65.00 [46.25, 71.75]	65.00 [46.25, 71.75]	50.00 [32.00, 69.00]
Gender	Male	22 (68.8)	12 (80.0)	10 (58.8)
Race	White	20 (62.5)	9 (60.0)	11 (64.7)
	Black	11 (34.4)	5 (33.3)	6 (35.3)
	Other	1 (3.1)	1 (6.7)	0 (0.0)
Hispanic		1 (3.1)	1 (6.7)	0 (0.0)
Past Medical History				
Hypertension		17 (53.1)	5 (33.3)	12 (70.6)
Diabetes		7 (21.9)	3 (20.0)	4 (23.5)
Chronic Kidney Disease		5 (15.6)	2 (13.3)	3 (17.6)
Congestive Heart Failure		6 (18.8)	3 (20.0)	3 (17.6)
Cancer		7 (21.9)	4 (26.7)	3 (17.6)
ESTOP at Enrollment		0.04 [0.03, 0.04]	0.04 [0.02, 0.04]	0.04 [0.04, 0.04]
Max ESTOP Prior to Enrollment		0.04 [0.03, 0.05]	0.03 [0.03, 0.05]	0.04 [0.03, 0.05]
ESTOP Risk Stratification Group	High risk (≥0.057)	8 (25.0)	4 (26.7)	4 (23.5)
	Moderate risk (0.01-0.0569)	24 (75.0)	11 (73.3)	13 (76.5)
Patient Location at Randomization	Ward/Floor	16 (50.0)	8 (53.3)	8 (47.1)
	ICU	16 (50.0)	7 (46.7)	9 (52.9)
Time from Admission to Enrollment (hours)		54.08 [41.56, 96.52]	42.35 [40.50, 90.50]	65.00 [43.00, 112.83]
Mechanical Ventilation at Enrollment		14 (43.8)	6 (40.0)	8 (47.1)
Foley Catheter at Enrollment		19 (59.4)	8 (53.3)	11 (64.7)
SOFA at Enrollment for ICU patients		4.00 [2.75, 6.25]	3.00 [1.50, 4.50]	5.00 [3.00, 7.00]
MEWS at Enrollment for Ward patients		3.00 [2.75, 4.00]	3.00 [2.75, 3.25]	3.00 [2.75, 4.00]
APACHE II at Enrollment for ICU patients		15.00 [12.00, 20.50]	12.50 [11.25, 14.50]	15.00 [12.00, 21.00]

© 2026 Churpek MM et al. *JAMA Network Open*.

Pre-Hospitalization Baseline SCr		1.24 (0.52)	1.13 (0.36)	1.33 (0.62)
Enrollment SCr		1.28 (0.51)	1.19 (0.42)	1.36 (0.59)
Enrollment Blood Urea Nitrogen		23.00 (18.45)	23.67 (24.23)	22.41 (12.04)
Race and Hispanic ethnicity were reported as recorded by the electronic health record. Other race includes Asian, Unknown and Mixed. All values reported as either No(%), Mean(SD) or median IQR				

Categorical variables are compared using the chi-squared test and summarized as counts and percentages; continuous variables are compared with the rank-sum test and summarized by medians and interquartile range.

© 2026 Churpek MM et al. *JAMA Network Open*.

eTable 18. Baseline Demographics and Characteristics of All Enrolled Patients Overall and Grouped by Treatment Arm: ENC (Early Nephrology Consult) and UC (Usual Care) Enrolled After the Spring 2020 COVID-19 Pandemic–Based Pause in Enrollment

		Overall	ENC	UC
n		148	74	74
Age		62.00 [51.75, 70.25]	60.00 [52.00, 69.75]	63.50 [50.75, 72.00]
Gender	Male	80 (54.1)	41 (55.4)	39 (52.7)
Race	White	75 (50.7)	34 (45.9)	41 (55.4)
	Black	63 (42.6)	33 (44.6)	30 (40.5)
	Other	10 (6.8)	7 (9.5)	3 (4.1)
Hispanic		5 (3.4)	3 (4.1)	2 (2.7)
Past Medical History				
Hypertension		87 (58.8)	45 (60.8)	42 (56.8)
Diabetes		40 (27.0)	26 (35.1)	14 (18.9)
Chronic Kidney Disease		7 (4.7)	3 (4.1)	4 (5.4)
Congestive Heart Failure		17 (11.5)	9 (12.2)	8 (10.8)
Cancer		39 (26.4)	18 (24.3)	21 (28.4)
ESTOP at Enrollment		0.01 [0.01, 0.02]	0.01 [0.01, 0.01]	0.01 [0.01, 0.02]
Max ESTOP Prior to Enrollment		0.01 [0.01, 0.02]	0.01 [0.01, 0.02]	0.02 [0.01, 0.03]
ESTOP Risk Stratification Group	High risk (≥0.057)	8 (5.4)	5 (6.8)	3 (4.1)
	Moderate risk (0.01-0.0569)	140 (94.6)	69 (93.2)	71 (95.9)
Patient Location at Randomization	Ward/Floor	63 (42.6)	33 (44.6)	30 (40.5)
	ICU	85 (57.4)	41 (55.4)	44 (59.5)
Time from Admission to Enrollment (hours)		64.94 [42.01, 114.47]	64.71 [41.88, 89.65]	65.17 [42.02, 115.37]
Mechanical Ventilation at Enrollment		23 (15.5)	12 (16.2)	11 (14.9)
Foley Catheter at Enrollment		69 (46.6)	29 (39.2)	40 (54.1)
SOFA at Enrollment for ICU patients		4.00 [3.00, 6.25]	4.00 [3.00, 6.00]	4.00 [3.00, 7.00]
MEWS at Enrollment for Ward patients		3.00 [2.00, 5.00]	3.00 [2.00, 4.00]	3.00 [2.00, 5.00]
APACHE II at Enrollment for ICU patients		16.50 [13.75, 21.25]	15.00 [12.50, 21.00]	18.00 [15.00, 21.00]

© 2026 Churpek MM et al. *JAMA Network Open*.

Pre-Hospitalization Baseline SCr		0.97 (0.40)	0.95 (0.36)	0.99 (0.45)
Enrollment SCr		1.02 (0.39)	1.00 (0.36)	1.04 (0.43)
Enrollment Blood Urea Nitrogen		23.0 (16.4)	22.7 (13.4)	23.2 (19.0)
Race and Hispanic ethnicity were reported as recorded by the electronic health record. Other race includes – Asian, Unknown and Mixed. All values reported as either No(%), Mean(SD) or median IQR				

Categorical variables are compared using the chi-squared test and summarized as counts and percentages; continuous variables are compared with the rank-sum test and summarized by medians and interquartile range.

© 2026 Churpek MM et al. *JAMA Network Open*.

eTable 19. Outcomes of Patients Grouped by Treatment Arm: ENC (Early Nephrology Consult) and UC (Usual Care) Enrolled Prior to the Spring 2020 COVID-19 Pandemic Enrollment Pause

Categorical variables are compared using the chi-squared test and summarized as counts and percentages; continuous variables are compared with the rank-sum test and summarized by medians and interquartile range.

		Overall	ENC	UC	P-value
n		32	15	17	
Peak SCr during first 7 days post enrollment		1.28 (0.51)	1.19 (0.42)	1.36 (0.59)	0.35
Peak Change in SCr from enrollment		1.44 (0.76)	1.35 (0.53)	1.52 (0.92)	0.55
Peak Change in SCr from pre-admission baseline		0.16 (0.45)	0.17 (0.36)	0.16 (0.53)	0.96
Dialysis		1 (3.1)	0 (0.0)	1 (5.9)	>0.99
Death		1 (3.1)	0 (0.0)	1 (5.9)	>0.99
Maximum KDIGO Stage	No AKI	19 (59.4)	10 (66.7)	9 (52.9)	0.73
	One	8 (25.0)	3 (20.0)	5 (29.4)	
	Two	5 (15.6)	2 (13.3)	3 (17.6)	
	Three	0 (0.0)	0 (0.0)	0 (0.0)	
Duration of All AKI (n=64)		14.75 (12.23)	13.33 (10.83)	16.00 (13.55)	0.55
Duration of Severe AKI (Stage 2 or 3) n=25		3.95 (2.53)	3.62 (2.33)	4.18 (2.75)	0.65
Hospital Length of Stay (Days)		1.28 (0.51)	1.19 (0.42)	1.36 (0.59)	0.35
ICU Length of Stay (Days)		1.44 (0.76)	1.35 (0.53)	1.52 (0.92)	0.55
SCr – serum creatinine (mg/dL) - All values reported as either No(%) or mean(SD)					

© 2026 Churpek MM et al. *JAMA Network Open*.

eTable 20. Outcomes of Patients Grouped by Treatment Arm: ENC (Early Nephrology Consult) and UC (Usual Care) Enrolled After the Spring 2020 COVID-19 Pandemic Enrollment Pause

Categorical variables are compared using the chi-squared test and summarized as counts and percentages; continuous variables are compared with the rank-sum test and summarized by medians and interquartile range.

		Overall	ENC	UC	P-value
n		148	74	74	
Peak SCr during first 7 days post enrollment		1.02 (0.39)	1.00 (0.36)	1.04 (0.43)	0.56
Peak Change in SCr from enrollment		1.04 (0.63)	1.06 (0.67)	1.03 (0.59)	0.71
Peak Change in SCr from pre-admission baseline		0.02 (0.47)	0.06 (0.53)	-0.02 (0.41)	0.28
Dialysis		4 (2.7)	3 (4.1)	1 (1.4)	0.61
Death		13 (8.8)	8 (10.8)	5 (6.8)	0.56
Maximum KDIGO Stage	No AKI	91 (61.5)	42 (56.8)	49 (66.2)	0.56
	One	33 (22.3)	17 (23.0)	16 (21.6)	
	Two	19 (12.8)	12 (16.2)	7 (9.5)	
	Three	5 (3.4)	3 (4.1)	2 (2.7)	
Duration of All AKI (n=64)		13.06 (15.01)	13.18 (15.10)	12.95 (15.03)	0.93
Duration of Severe AKI (Stage 2 or 3) n=25		5.55 (6.12)	5.98 (6.35)	5.14 (5.94)	0.54
Hospital Length of Stay (Days)		1.02 (0.39)	1.00 (0.36)	1.04 (0.43)	0.56
ICU Length of Stay (Days)		1.18 (0.60)	1.22 (0.65)	1.15 (0.56)	0.52
SCr – serum creatinine (mg/dL) - All values reported as either No(%) or mean(SD)					

© 2026 Churpek MM et al. *JAMA Network Open*.

eFigure. Serum Creatinine Over Time—Average Preadmission Baseline SCr (Base) as Well as the Enrollment (Enroll) and Subsequent 7-Day of SCr for Patients in the ENC and UC Study Arms

There was no significant difference between the two arms of the study (Wilcoxon rank-sum test p>0.05 for all time points).

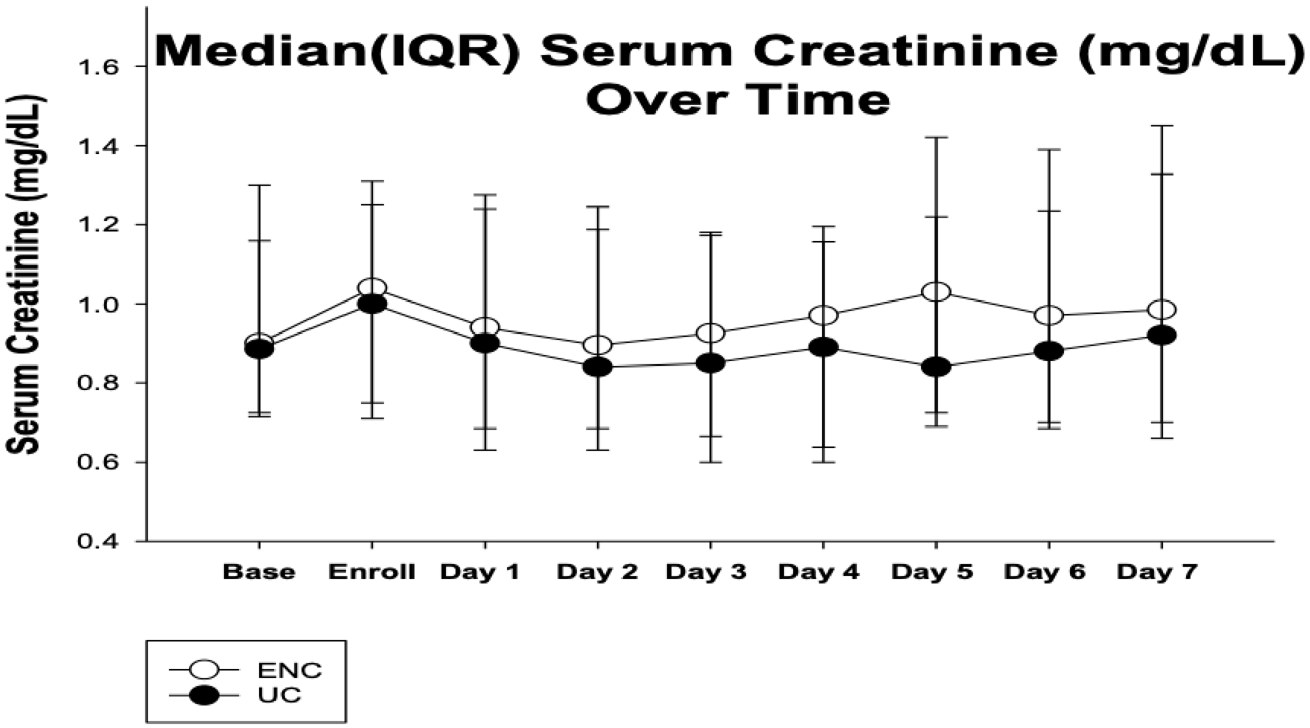

© 2026 Churpek MM et al. *JAMA Network Open*.
