# Supplementary material for: Early Nephrology Consultation and Acute Kidney Injury in Hospitalized Patients: A Randomized Clinical Trial
Source: JAMA Netw Open. 2026 Jul 10;9(7):e2622554. doi: 10.1001/jamanetworkopen.2026.22554 (PMC13355147; doi:10.1001/jamanetworkopen.2026.22554)
Supplement: Supplement 3. — Nonauthor Collaborators [file jamanetwopen-e2622554-s003.pdf]

Supplemental Online Content: Nonauthor Collaborators
*First name, last name, and suffix (if applicable) are required and will appear in PubMed.

***Group Name(s): E-STOP Collaborators**							
***First Name and Middle Initial(s)**	***Last Name**	***Suffix (eg, Jr, III)**	Academic Degrees	Institution	Location (city, state/province, country)	Role or Contribution, eg, chair, principal investigator	Group (if more than 1 Group listed in the byline) and/or Subgroup (eg, Steering Committee)
Katherine	Chen			University of Chicago	Chicago, IL , USA	Research Assistant	1
Melody	Dias		BA	University of Chicago	Chicago, IL , USA	Research Assistant	1
Joshua	Gonzalez			University of Chicago	Chicago, IL , USA	Research Assistant	1
Riley	Hamilton		BA	University of Chicago	Chicago, IL , USA	Research Assistant	1
Alissa	Kunczt		BA	University of Chicago	Chicago, IL , USA	Research Assistant	1
Christina	Li		BA	University of Chicago	Chicago, IL , USA	Research Assistant	1
Kelly	Minna		BA	University of Chicago	Chicago, IL , USA	Research Assistant	1
Sharon	Trevino		RN	University of Chicago	Chicago, IL , USA	Research Nurse	1
