# Supplementary material for: Early Nephrology Consultation and Acute Kidney Injury in Hospitalized Patients: A Randomized Clinical Trial
Source: JAMA Netw Open. 2026 Jul 10;9(7):e2622554. doi: 10.1001/jamanetworkopen.2026.22554 (PMC13355147; doi:10.1001/jamanetworkopen.2026.22554)
Supplement: Supplement 4. — Data Sharing Statement [file jamanetwopen-e2622554-s004.pdf]

Data Sharing Statement

Churpek. Early Nephrology Consultation and Acute Kidney Injury in Hospitalized Patients at Risk. *JAMA Netw Open*. Published July 10, 2026. doi:10.1001/jamanetworkopen.2026.22554

Data
Additional Information: https://clinicaltrials.gov/study/NCT03590028
Data available: Yes
Data types: Deidentified participant data
How to access data: individual patient data will be deidentified and shared per Univeristy of Chicago protocol
When available: With publication

Supporting Documents
Document types: None

Additional Information
Who can access the data: those whose proposed use has been approved
Types of analyses: those whose proposed use has been approved
Mechanisms of data availability: signed data use agreement
